# Why base-catalyzed isomerization of *N*-propargyl amides yields mostly allenamides rather than ynamides

**DOI:** 10.3762/bjoc.11.156

**Published:** 2015-08-18

**Authors:** Armando Navarro-Vázquez

**Affiliations:** 1 Departamento de Química Fundamental, Centro de Ciências Exatas e da Natureza, Universidade Federal de Pernambuco, Cidade Universitária - Recife, PE - CEP 50.740-560, Brazil

**Keywords:** allenamide, DFT, isomerization, ynamide

## Abstract

The base-catalyzed isomerization of *N*-propargylamides or carbamates may furnish *N*-allenyl compounds (allenamides/allencarbamates) or further evolve to *N*-alkynyl compounds (ynamides or yncarbamates). The particular fate of this reaction varies from experiment to experiment and there is no clear rule for predicting the reaction outcome for a particular structure. With the support of ab initio and DFT computations, this work shows that observed results can be explained by assuming an exchange equilibrium between energetically close *N*-propargyl, allenyl and *N*-alkynyl forms and that the reaction outcome correlates to a particular equilibrium mixture. Due to the very small energy gap between the *N*-allenyl and *N*-alkynyl forms, small structural changes may easily alter the equilibrium position, explaining the variety of observed experimental results. Based on CBS-QB3 computations, the ωB97 functional provided reasonably accurate isomerization energies and could successfully predict the experimentally observed behavior for several examples from the literature.

## Introduction

**Scheme 1:**
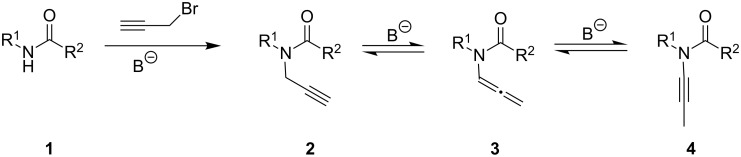
Preparation of propargylamides through alkylation of secondary amides and base-catalyzed isomerization to allenamides and ynamides.

Allenamides [1–2] and ynamides [3–5] have become useful functional groups for organic chemistry synthesis in the last years. Reduced electron pair donation as compared to their enamine or ynamine parents strongly improves the handling of these compounds by making them resistant to hydrolysis while still retaining a rich variety of chemistry. The simplest method for the preparation of these compounds is the alkylation of a secondary amide **1** with a propargyl bromide (Scheme 1) [6–7]. Subsequent base-catalyzed isomerization of *N*-propargyl compound **2** leads to allenamide **3**. Whereas in some particular cases the reaction leads to the final *N*-alkynyl product, in most cases, the reaction stops at the allene stage and does not further progress even when employing harsh reaction conditions. The outcome of the isomerization process seems to subtlety depend on the structural features of the *N*-propargyl compound. Similar issues have been observed for carbamates or phosphoramides [8].

In summary, the isomerization process is not yet well understood and, quoting Hsung and coworkers, “…This poses an interesting fundamental question as to why the thermodynamically more stable ynamide … was not found if these isomerizations involved an equilibration mechanism…” [2].

The available experimental data strongly suggest that allenamides do not always convert to ynamides due to thermodynamic rather than kinetic reasons, and it is not necessarily the case that ynamides are more stable than the parent allenamides. Here it is hypothesized that *N*-propargylamide ↔ allenamide ↔ ynamide interconversion takes place reversibly and the outcome of the reaction is related to the equilibrium position. Structural changes can alter the equilibrium position, leading to the observation of either species or a mixture thereof [8].

**Figure 1:**
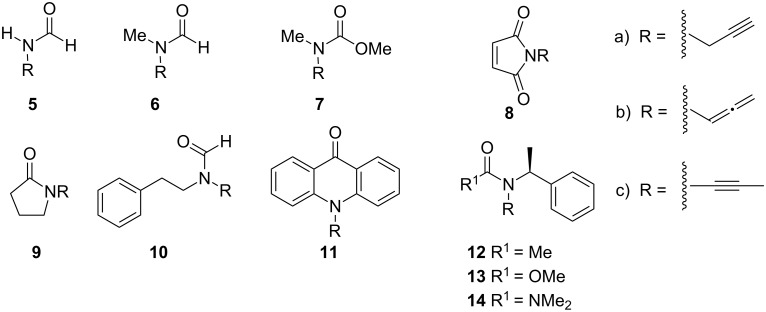
Set of studied compounds.

This hypothesis states that the course of the reaction in Scheme 1 simply reflects the relative energy of isomers **2**, **3** and **4**. To test this, the relative energies for the corresponding isomers for a selected combination of starting amides or carbamates and a final urea example (Figure 1) were computed using ab initio and DFT methods.

## Results and Discussion

### Computational procedures

It is known that ab initio prediction of cumulene–polyynes isomerization energies requires a very high level of theory [9], and expensive CCSD(T)/cc-pVQZ//MP2/cc-pVTZ coupled cluster computations were needed to match the experimental propadiene to propyne isomerization energy of −1.4 kcal/mol [10]. In order to choose a standard test methodology for the computation of allenamide–ynamide relative energies, the performance of the composite CBS-QB3 [11–12] methodology on the propadiene to propyne isomerization process as well in the simplest allenamide to ynamide isomerization reactions in compound **5** was evaluated. For the sake of simplicity, the conformation of the amide bond in **5a**,**b**,**c** was fixed as s-*E*. This CBS-QB3 method furnished a Δ*H*_0_ energy of −0.8 kcal/mol for propadiene to propyne isomerization, which is somewhat lower than the reported experimental energy. Note, however, that the result is close to that furnished by the much more computationally expensive W1 [13] computation on the same system with a Δ*H*_0_ value of −1.1 kcal/mol (Table 1).

**Table 1:** Isomerization energies for the propadiene to propyne and the **5a**,**b**,**c** system.

Level	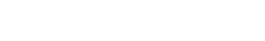	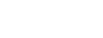	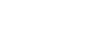

CBS-QB3	−0.8	−4.3	1.0
W1	−1.1	–	–
M05	0.5	−6.6	0.3
M052X	1.6	−6.8	2.8
M06L	2.3	−9.3	2.3
M06HF	0.6	−5.0	2.4
M06	0.6	−6.5	0.5
M062X	0.6	−6.0	1.3
ωΒ97	0.4	−5.3	1.1
ωB97X	1.0	−6.2	1.8
ωB97XD	1.9	−7.2	2.9
B3LYP	3.0	−9.2	3.8

The best performing DFT functional for the prediction of cumulene–polyynes isomerization energies was found by Zhao and Truhlar to be the hybrid meta-GGA M05 functional [9]. Since then, new functionals of the same family have been reported such as the M06 set [14] or the ωB97 family of long-range corrected GGAs [15]. In this work, these new functionals were tested to determine if they could provide superior performance for the given problem. In the M06 family, the non-hybrid M06L was tested, as well as the hybrid M06HF and M062X forms. From the ωB97 family we tested the ωB97 form, the ωB97X, which includes exact short-range HF exchange, and in addition the ωB97XD functional, which also includes Grimme’s dispersion correction [16]. The DFT tests employed a medium-size 6-31+G** basis in search of a methodology applicable to relatively large systems. The relative energies for the propadiene–propyne isomerization process as well as for the **a**,**b**,**c** isomers of compound **5** were computed.

Although all functionals predicted the wrong sign for the propadiene to propyne reaction M05, M062X and ωB97 provided the closest isomerization energies (Table 1). B3LYP, as previously reported by Truhlar [9], and the non-hybrid GGA M06L functional presented particularly bad performance.

In the case of the **5a**,**b**,**c** system, the CBS-QB3 computations predicted the *N*-propargyl to allenamide 
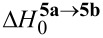
 isomerization to be a moderately exothermic reaction with a value of −4.3 kcal/mol (Table 1). As compared to CBS-QB3, all the DFT computations overestimated the exothermicity of this reaction by more than 1.5 kcal/mol save for the M06HF and ωB97 functionals with 
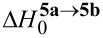
 values of −5.0 and −5.3 kcal/mol, respectively. Particularly inadequate was the performance of M06L and B3LYP with Δ*H*_0_ values far off by around 5 kcal/mol. For the reaction of interest, the allenamide–ynamide conversion, CBS-QB3 predicted a small energy gap between the two forms with a positive 
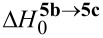
 value of 1.0 kcal/mol. Here M062X and ωB97 performed best with values very close to the CBS-QB3 ones. Overall, the ωB97 functional offered the best performance and was the chosen functional for the investigation of the larger systems.

The next investigated system was the *N*-methylformamide **6** (again the conformation of the peptidic bond was fixed as *s*-*E*). Both CBS-QB3 and ωB97 predicted the allenamide **6b** to be notably more stable than the *N*-propargyl compound **6a** (Table 2). On the other hand, the ynamide **6c** is more than 2 kcal/mol larger than the allenamide compound. The effect of solvation, which can be important in the polar solvents where the isomerization reaction is commonly carried out, was considered at the PCM level [17] by using DMSO parameters. The DFT PCM computations showed reduced exothermicity of the *N*-propargylamide → allenamide step and increased endothermicity of the allenamide → ynamide gap (Table 2). Hence, the results were in agreement with the experimental observation that base-catalyzed isomerization of most *N*-propargylamides commonly furnish allenamide compounds, and clearly show that simple ynamides are not inherently more stable than allenamides.

**Table 2:** Isomerization energies for the amides/carbamates outlined in Figure 1.

	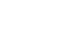			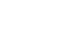		

	CBS-QB3	ωB97		CBS-QB3	ωB97	

**5**	−4.3	−5.3^a^	−3.6^b^	1.0	1.1^a^	1.8^b^
**6**	−4.9	−5.7	−4.1	2.4	2.3	3.0
**7**	−4.6	−5.5	−3.9	3.1	3.4	2.6
**8**	−1.3	−2.4	−1.2	3.2	3.7	2.5
**9**	–	−5.5	−4.2	–	4.8	3.1
**10**	–	−5.3	−4.0	–	2.6	2.8
**11**	–	−3.3	−2.7	–	−2.3	−1.1
**12**	–	–	−2.2	–	–	−1.7
**13**	–	–	−2.3	–	–	0.0
**14**	–	–-	−4.6	–	–	−1.2

^a^In vacuum; ^b^PCM(DMSO).

CBS-QB3 and ωB97 computations on the *N*-methylcarbamate system **7**, also with a fixed *s*-*E* conformation, showed a similar pattern to that computed for amide **6**. Note, however, that whereas inclusion of solvation increases the allenamide → ynamide gap, the allencarbamate → yncarbamate gap is decreased from 3.4 to 2.6 kcal/mol (Table 2).

According to Hsung and coworkers, *N*-propargylphthalimide does not lead to either allenamides or ynamides under typical isomerization conditions [18]. At the CBS-QB3 level, the *N*-propargylimide **8a** is only 1.3 kcal/mol more stable than the corresponding allenamide **8b**. The now lower exothermicity of the reaction may account for the observed lack of reactivity since entropic and solvation effects could make the propargyl amide ynamide transformation endergonic. In fact, the ωB97 reaction energy is more than 1 kcal/mol less exothermic when computed in DMSO than in vacuum. Note that in any case the 
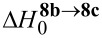
 ωB97 reaction energy indicates that the reaction would very likely stop at the allenamide stage (Table 2). Nonetheless, the above results suggest that varying the reaction conditions (perhaps solvent polarity) could eventually lead to the observation of the allenimides. NMR deuteration studies could be of help to experimentally eliminate possible kinetic effects in the observed lack of reactivity [19].

Having established a reasonable performance of ωB97 in describing the above systems, the reproducibility of experimental observations in larger systems was tested. The isomerization of lactam **9** was first studied. The isomerization of *N*-propargyllactams is known to stop at the allenamide stage [20–21]. The computations showed that the allenamide is much more stable than the *N*-propargyl compound (
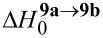
 = −5.5 and −4.2 kcal/mol) or the ynamide (
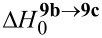
= 4.8 and 3.1 kcal/mol), both in vacuum and DMSO, which is in agreement with the experimental observations (Table 2). Similar results, as can be seen in Table 2, were obtained for the *N*-(2-phenethyl)formamide system **10**, which was prepared during previous studies on acid-catalyzed cyclization of allenamides [22].

The isomerization of acridinone is a frequently studied process and one of the few examples where isomerization continues to the final ynamide stage [23–24]. Interestingly, the kinetics of the reaction seem to be slow enough to allow the allenamide intermediate to be obtained by simply employing a short reaction time [20]. The computations for acridinone **11** gave isomerization energies 
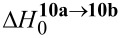
 and 
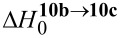
 of −3.3 and −2.3 kcal/mol, in the gas phase, and −2.7 and −1.1 in DMSO, respectively. This agrees with the experimental observation of the allenamide further evolving to the ynamide product. The now lower energy of the alkyne system can be attributed to the distortion of the allenamide moiety from planarity due to strong steric interactions with the hydrogen atoms in the ortho position.

**Figure 2:**
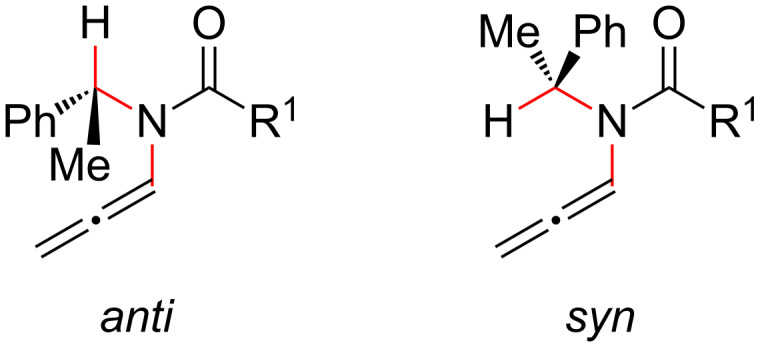
*Anti* and *syn* conformations around the N–C=C=C bond for *N*-allenyl compounds **12b**–**14b**.

A final and more demanding test is the Hsung observation that whereas only ynamide **12** was observed upon isomerization of the corresponding *N*-propargylamide, the related carbamate **13** only evolves to the allenamide stage [18]. Furthermore, urea **14** (see page 5069 of [3], we thank one of the referees for highlighting this example) again furnished only the ynamide compound. The ωB97 computations predict the ynamide **12c** to be more stable than allenamide **12b** by 1.7 kcal/mol (Table 2). On the other hand, the yncarbamate **13c** is nearly degenerate with the allencarbamate **13b**. For the urea **14** this trend is again reverted and the *N*-alkynyl compound **14c** is more stable than the allenamide **14b** by 1.2 kcal/mol. Note the more exothermic *N*-propargyl → *N*-allenyl gap for **14** caused by the higher electronic donation capability of the nitrogen atom. Hence, the DFT computations are in agreement with the observed reaction trends. This could be potentially explained in terms of the destabilization of the allenamide form due to steric crowding as suggested by a less exothermic *N*-propargyl → *N*-allenyl gap. This effect is large enough to result in the experimental observation of the ynamides **12c** and ynurea **14c**, but not the yncarbamate **13c**. The difference in the *N*-allenyl → *N*-alkynyl gap for **12** and **13** could be partially attributed to the different basal conformations adopted by allenes **12b** and **13b** whereas the methynic proton in the benzylic chain respectively prefers an *anti* or *syn* conformation with respect to the N–C=C= bond (Figure 2). The *anti* conformation is also the one preferred by the allenyl urea **14b**.

## Conclusion

In summary, the very different outcome of the isomerization reaction of *N*-propargylamides and carbamates towards allenamides or ynamides can be simply explained in terms of the relative energy of the involved species. Simple allenamides or allencarbamates are in general more stable than the corresponding *N*-alkynyl isomers but the sign of the small energy gap can be reversed through structural changes. The ωB97 functional gave reasonable performance for the computation of isomerization energies, providing a suitable computational methodology for the prediction of the result of the isomerization reaction in similar systems.

## Computational Procedures

All reaction energies are reported as enthalpies at zero K (Δ*H_0_* = Δ*E* + ZPVE). The structures were fully optimized at the reported levels of theory. The DFT computations used a pruned (99,590) grid (Gaussian09 ultrafine grid). The analytical frequencies were computed to verify the nature as minima of all computed structures and to obtain the zero-point vibrational energies. The zero-point vibrational energies are unscaled in the DFT computations and scaled according to method-defined factors in CBS-QB3 [11–12] and W1 [13] computations. The DFT PCM [17] computations were performed using DMSO Gaussian09 parameters. All computations were performed using the Gaussian09 package [25].

For species **9**, **10, 12, 13** and **14** a conformational search was accomplished using the GMMX method and the MMX force field as implemented in PCModel 9.21 [26]. The five lowest-energy conformations were then selected and the DFT was optimized at the ωB97/PCM(DMSO) level. The structure with the lowest DFT energy was then selected for computation of isomerization energies.

## Supporting Information

File 1 Computational procedures as well as energies and XYZ coordinates for all computed structures.

## References

[1] Lu T, Lu Z, Ma Z-X, Zhang Y, Hsung R P (2013). Chem Rev.

[2] Wei L-L, Xiong H, Hsung R P (2003). Acc Chem Res.

[3] DeKorver K A, Li H, Lohse A G, Hayashi R, Lu Z, Zhang Y, Hsung R P (2010). Chem Rev.

[4] Evano G, Coste A, Jouvin K (2010). Angew Chem, Int Ed.

[5] Wang X-N, Yeom H-S, Fang L-C, He S, Ma Z-X, Kedrowski B L, Hsung R P (2013). Acc Chem Res.

[6] Dickinson W B, Lang P C (1967). Tetrahedron Lett.

[7] Bousfield T W, Kimber M C (2015). Tetrahedron Lett.

[8] Corbel B, Paugam J-P, Dreux M, Savignac P (1976). Tetrahedron Lett.

[9] Zhao Y, Truhlar D G (2006). J Phys Chem A.

[10] Lee Woodcock H F, Schaefer A, Schreiner P R (2002). J Phys Chem A.

[11] Montgomery J A, Frisch M J, Ochterski J W, Petersson G A (1999). J Chem Phys.

[12] Montgomery J A, Frisch M J, Ochterski J W, Petersson G A (2000). J Chem Phys.

[13] Parthiban S, Martin J M L (2001). J Chem Phys.

[14] Zhao Y, Truhlar D G (2008). Theor Chem Acc.

[15] Chai J-D, Head-Gordon M (2008). J Chem Phys.

[16] Grimme S (2006). J Comput Chem.

[17] Tomasi J, Mennucci B, Cammi R (2005). Chem Rev.

[18] Huang J, Xiong H, Hsung R P, Rameshkumar C, Mulder J A, Grebe T P (2002). Org Lett.

[19] Bew S P, Hiatt-Gipson G D, Lovell J A, Poullain C (2012). Org Lett.

[20] Wei L-L, Mulder J A, Xiong H, Zificsak C A, Douglas C J, Hsung R P (2001). Tetrahedron.

[21] Fenández I, Monterde M I, Plumet J (2005). Tetrahedron Lett.

[22] Navarro-Vázquez A, Rodríguez D, Martínez-Esperón M F, García A, Saá C, Domínguez D (2007). Tetrahedron Lett.

[23] Katritzky A R, Ramer W H (1985). J Org Chem.

[24] Mahamoud A, Galy J P, Vincent E J, Barbe J (1981). Synthesis.

[25] (2009). Gaussian 09.

[R26] 26Serena Software Box 3076 Bloomington, IN 47402-3076.

