# Supplementary material for: Why base-catalyzed isomerization of N-propargyl amides yields mostly allenamides rather than ynamides
Source: Beilstein J Org Chem. 2015 Aug 18;11:1441–6. doi: 10.3762/bjoc.11.156 (PMC4578395; doi:10.3762/bjoc.11.156)
Supplement: File 1 — Computational procedures as well as energies and XYZ coordinates for all computed structures. [file Beilstein_J_Org_Chem-11-1441-s001.pdf]

Supporting Information

for

Why base catalyzed isomerization of *N*-propargyl amides yields mostly allenamides rather than ynamides

Armando Navarro-Vázquez

Address: Departamento de Química Fundamental, Centro de Ciências Exatas e da Natureza, Universidade Federal de Pernambuco, Cidade Universitária - Recife, PE - CEP 50.740-560, Brazil

Email: Armando Navarro-Vázquez - armando.deus@gmail.com

Computational procedures as well as energies and XYZ coordinates for all computed structures

Computed energies and XYZ coordinates (Å)

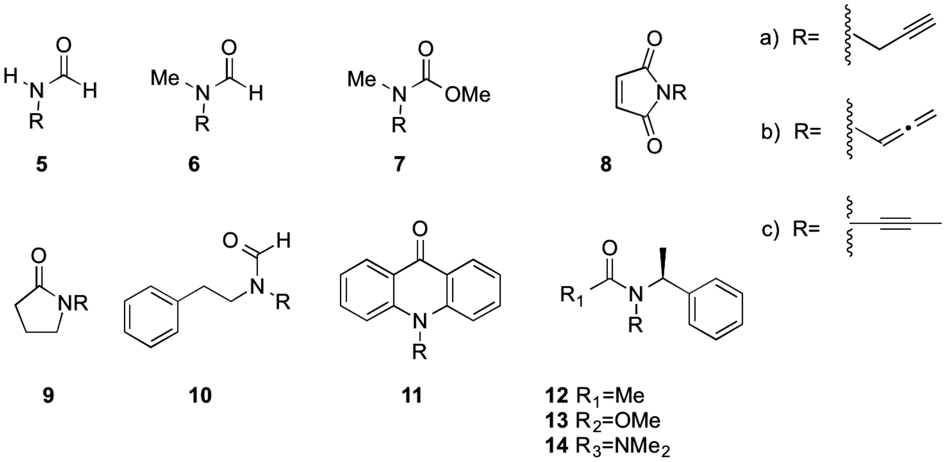

Propadiene

CBS-QB3
E(0 K)= -116.42037 a.u.

```
7
propadiene CBS-QB3
C      0.000000    0.000000    1.302534
C      0.000000    0.000000    0.000000
C      0.000000    0.000000   -1.302534
H      0.000000    0.927184    1.866962
H      0.000000   -0.927184    1.866962
H     -0.927184    0.000000   -1.866962
H      0.927184    0.000000   -1.866962
```

W1
E(0 K)= -116.62670 a.u.

```
7
propadiene W1
C      0.000000    0.000000    1.300052
C      0.000000    0.000000    0.000000
C      0.000000    0.000000   -1.300052
H      0.000000    0.925351    1.863005
H      0.000000   -0.925351    1.863005
H     -0.925351    0.000000   -1.863005
H      0.925351    0.000000   -1.863005
```

M05
E(SCF)= -116.56500 **a.u.**
E(SCF+ZPVE)= -116.50985 **a.u.**

```
7
propadiene M05
C      0.000000    0.000000    1.309739
C      0.000000    0.000000    0.000000
C      0.000000    0.000000   -1.309739
H      0.000000    0.930487    1.870783
H      0.000000   -0.930487    1.870783
H     -0.930487    0.000000   -1.870783
H      0.930487    0.000000   -1.870783
```

M052X
E(SCF)= -116.64304 a.u.
E(SCF+ZPVE)= -116.58684 a.u.

```
7
propadiene M052X
C      0.000000    0.000000    1.306857
C      0.000000    0.000000    0.000000
C      0.000000    0.000000   -1.306857
H      0.000000    0.930334    1.862332
H      0.000000   -0.930334    1.862332
H     -0.930334    0.000000   -1.862332
H      0.930334    0.000000   -1.862332
```

M06L
E(SCF)= -116.64464 a.u.
E(SCF+ZPVE)= -116.58967 a.u.

```
7
propadiene M06L
C      0.000000    0.000000    1.306124
C      0.000000    0.000000    0.000000
C      0.000000    0.000000   -1.306124
H      0.000000    0.927664    1.871905
H      0.000000   -0.927664    1.871905
H     -0.927664    0.000000   -1.871905
H      0.927664    0.000000   -1.871905
```

M06HF
E(SCF)= -116.62525 a.u.
E(SCF+ZPVE)= -116.56840 a.u.

```
7
propadiene M06HF
C      0.000000    0.000000    1.308022
C      0.000000    0.000000    0.000000
C      0.000000    0.000000   -1.308022
H      0.000000    0.932071    1.859151
H      0.000000   -0.932071    1.859151
H     -0.932071    0.000000   -1.859151
H      0.932071    0.000000   -1.859151
```

M06
E(SCF)= -116.56640 a.u.
E(SCF+ZPVE)= -116.51159 a.u.

```
7
propadiene M06
C      0.000000    0.000000    1.305646
C      0.000000    0.000000    0.000000
C      0.000000    0.000000   -1.305646
H      0.000000    0.929870    1.870846
H      0.000000   -0.929870    1.870846
H     -0.929870    0.000000   -1.870846
H      0.929870    0.000000   -1.870846
```

M062X
E(SCF)= -116.60306 a.u.
E(SCF+ZPVE)= -116.54733 a.u.

```
7
propadiene M062X
C      0.000000    0.000000    1.307192
C      0.000000    0.000000    0.000000
C      0.000000    0.000000   -1.307192
H      0.000000    0.931123    1.865448
H      0.000000   -0.931123    1.865448
H     -0.931123    0.000000   -1.865448
H      0.931123    0.000000   -1.865448
```

WB97
E(SCF)= -116.63180 a.u.

E(SCF+ZPVE)= -116.57574 a.u.

```
7
propadiene WB97
C      0.000000    0.000000    1.309396
C      0.000000    0.000000    0.000000
C      0.000000    0.000000   -1.309396
H      0.000000    0.933954    1.867642
H      0.000000   -0.933954    1.867642
H     -0.933954    0.000000   -1.867642
H      0.933954    0.000000   -1.867642
```

WB97X
E(SCF)= -116.62650 a.u.
E(SCF+ZPVE)= -116.57048 a.u.

```
7
propadiene WB97X
C      0.000000    0.000000    1.307029
C      0.000000    0.000000    0.000000
C      0.000000    0.000000   -1.307029
H      0.000000    0.931735    1.865715
H      0.000000   -0.931735    1.865715
H     -0.931735    0.000000   -1.865715
H      0.931735    0.000000   -1.865715
```

WB97XD
E(SCF)= -116.62102 **a.u.**
E(SCF+ZPVE)= -116.56528 **a.u.**

```
7
propadiene WB97XD
C      0.000000    0.000000    1.306714
C      0.000000    0.000000    0.000000
C      0.000000    0.000000   -1.306714
H      0.000000    0.930858    1.866146
H      0.000000   -0.930858    1.866146
H     -0.930858    0.000000   -1.866146
H      0.930858    0.000000   -1.866146
```

propyne

CBS-QB3
E(0 K)=-116.42168 a.u.

```
7
propyne CBS-QB3
C      0.000000    0.000000    1.420033
C      0.000000    0.000000    0.219037
C      0.000000    0.000000   -1.237554
H      0.000000    0.000000    2.482009
H      0.000000    1.020583   -1.630368
H     -0.883851   -0.510292   -1.630368
H      0.883851   -0.510292   -1.630368
```

W1
E(0 K)= -116.62846 a.u.

```
7
propyne W1
C      0.000000    0.000000    1.417629
C      0.000000    0.000000    0.218596
C      0.000000    0.000000   -1.235649
H      0.000000    0.000000    2.478279
H      0.000000    1.018453   -1.627244
H     -0.882006   -0.509227   -1.627244
H      0.882006   -0.509227   -1.627244
```

M05
E(SCF)= -116.56472   a.u.
E(SCF+ZPVE)= -116.50901 a.u.

```
7
propyne M05
C      0.000000    0.000000    1.427223
C      0.000000    0.000000    0.213880
C      0.000000    0.000000   -1.241230
H      0.000000    0.000000    2.494746
H      0.000000    1.021250   -1.631328
H     -0.884428   -0.510625   -1.631328
H      0.884428   -0.510625   -1.631328
```

M052X
E(SCF)= -116.640922 a.u.
E(SCF+ZPVE)= -116.58422 a.u.

```
7
propyne M052X
C      0.000000    0.000000    1.424721
C      0.000000    0.000000    0.218205
C      0.000000    0.000000   -1.244730
H      0.000000    0.000000    2.490005
H      0.000000    1.021577   -1.626393
H     -0.884711   -0.510788   -1.626393
H      0.884711   -0.510788   -1.626393
```

M06L
E(SCF)=   -116.64156 a.u.
E(SCF+ZPVE)= -116.58603 a.u.

```
7
propyne M06L
C      0.000000    0.000000    1.423705
C      0.000000    0.000000    0.212751
C      0.000000    0.000000   -1.235210
H      0.000000    0.000000    2.488605
H      0.000000    1.019432   -1.632028
H     -0.882854   -0.509716   -1.632028
H      0.882854   -0.509716   -1.632028
```

M06HF
E(SCF)= -116.62479 a.u.

E(SCF+ZPVE)= -116.56747 a.u.

```
7
propyne M06HF
C      0.000000    0.000000    1.426862
C      0.000000    0.000000    0.223342
C      0.000000    0.000000   -1.254321
H      0.000000    0.000000    2.493165
H      0.000000    1.025125   -1.622821
H     -0.887784   -0.512562   -1.622821
H      0.887784   -0.512562   -1.622821
```

M06
E(SCF)= -116.56600 a.u.
E(SCF+ZPVE)= -116.51058 a.u.

```
7
propyne M06
C      0.000000    0.000000    1.423712
C      0.000000    0.000000    0.214801
C      0.000000    0.000000   -1.237477
H      0.000000    0.000000    2.490887
H      0.000000    1.021755   -1.632368
H     -0.884866   -0.510877   -1.632368
H      0.884866   -0.510877   -1.632368
```

M062X
E(SCF)= -116.60273 a.u.
E(SCF+ZPVE)= -116.546443 a.u.

```
7
propyne M062X
C      0.000000    0.000000    1.425096
C      0.000000    0.000000    0.218289
C      0.000000    0.000000   -1.244128
H      0.000000    0.000000    2.491966
H      0.000000    1.022729   -1.629169
H     -0.885710   -0.511365   -1.629169
H      0.885710   -0.511365   -1.629169
```

WB97
E(SCF)= -116.63173 **a.u.**
E(SCF+ZPVE)= -116.57513 **a.u.**

```
7
propyne WB97
C      0.000000    0.000000    1.427337
C      0.000000    0.000000    0.219743
C      0.000000    0.000000   -1.247242
H      0.000000    0.000000    2.497606
H      0.000000    1.025096   -1.632210
H     -0.887759   -0.512548   -1.632210
H      0.887759   -0.512548   -1.632210
```

WB97X
E(SCF)= -116.62531 a.u.
E(SCF+ZPVE)= -116.56881 a.u.

```
7
WB97X
C      0.000000    0.000000    1.424864
C      0.000000    0.000000    0.218647
C      0.000000    0.000000   -1.243547
H      0.000000    0.000000    2.492506
H      0.000000    1.022560   -1.630764
H     -0.885563   -0.511280   -1.630764
H      0.885563   -0.511280   -1.630764
```

WB97XD
E(SCF)= -116.61839 a.u.
E(SCF+ZPVE)= -116.56224 a.u.

7

propyne WB97XD

C	0.000000	0.000000	1.306714
C	0.000000	0.000000	0.000000
C	0.000000	0.000000	-1.306714
H	0.000000	0.930858	1.866146
H	0.000000	-0.930858	1.866146
H	-0.930858	0.000000	-1.866146
H	0.930858	0.000000	-1.866146

5
<u>5a</u>

CBS-QB3
E(0 K) = -284.88223 a.u.

```
11
5a CBS-QB3
N      0.560249    0.348651   -0.447269
C     -0.674473    0.924552    0.064535
C      1.604789   -0.045964    0.339729
O      2.587810   -0.628341   -0.055220
H      1.459955    0.250039    1.397958
C     -1.808470   -0.005317    0.061434
C     -2.738185   -0.764425    0.033690
H      0.587922    0.041571   -1.410382
H     -0.935016    1.816578   -0.514849
H     -0.482117    1.266653    1.086117
H     -3.556932   -1.441745    0.017474
```

M05
E(SCF)= -285.176781 a.u.
E(SCF+ZPVE)= -285.09274 a.u.

```
11
5a M05
N      0.556058    0.326813   -0.439113
C     -0.668401    0.919479    0.054123
C      1.608971   -0.041528    0.342547
O      2.598917   -0.620364   -0.057140
H      1.474061    0.261786    1.399616
C     -1.809666    0.000999    0.056906
C     -2.757386   -0.754710    0.040690
H      0.589139    0.013902   -1.399425
H     -0.917296    1.805209   -0.540501
H     -0.482664    1.274804    1.072659
H     -3.588089   -1.425916    0.032962
```

M052X
E(SCF)= -285.31252 a.u.
E(SCF+ZPVE)= -285.22704 a.u.

```
11
5a M052X
N      0.567260    0.396929   -0.440863
C     -0.666403    0.944457    0.088072
C      1.573042   -0.068555    0.345023
O      2.557493   -0.645636   -0.070106
H      1.418426    0.149043    1.414476
C     -1.788270   -0.007809    0.056191
C     -2.706539   -0.787854    0.013746
H      0.613357    0.144072   -1.416842
H     -0.938422    1.842666   -0.468626
H     -0.481094    1.252251    1.119078
H     -3.514007   -1.482883   -0.019387
```

M06L
E(SCF)= -285.31587 a.u.
E(SCF+ZPVE)= -285.23237 a.u.

```
11
5a M06L
N      0.545253    0.260661   -0.459229
C     -0.663865    0.898614    0.023267
C      1.622244   -0.006367    0.330524
O      2.617967   -0.605335   -0.025308
H      1.491807    0.410420    1.353395
C     -1.818765    0.011342    0.063101
C     -2.779049   -0.724965    0.070176
H      0.547517   -0.142561   -1.386184
H     -0.896987    1.779702   -0.588146
```

```
H     -0.446179    1.277780    1.029651
H     -3.620062   -1.379034    0.085942
```

M06HF
E(SCF)= -285.26142 a.u.
E(SCF+ZPVE)= -285.17515 a.u.

```
11
5a M06HF
N      0.582964    0.477310   -0.414953
C     -0.667234    0.975222    0.125584
C      1.536799   -0.116703    0.349270
O      2.519329   -0.664212   -0.097210
H      1.347546   -0.040610    1.427576
C     -1.769376   -0.017830    0.048202
C     -2.657688   -0.825422   -0.023322
H      0.670212    0.346544   -1.415462
H     -0.959953    1.876052   -0.411440
H     -0.506417    1.245205    1.169521
H     -3.441775   -1.546260   -0.086245
```

M06
E(SCF)= -285.17077 a.u.
E(SCF+ZPVE)= -285.08734 a.u.

```
11
5a M06
N      0.547187    0.278742   -0.452064
C     -0.664254    0.907592    0.032663
C      1.616325   -0.018786    0.336220
O      2.607542   -0.608322   -0.036875
H      1.487661    0.356091    1.375008
C     -1.815137    0.008289    0.060009
C     -2.767299   -0.734800    0.061332
H      0.568951   -0.090785   -1.395769
H     -0.900975    1.791628   -0.574819
H     -0.459135    1.281619    1.045082
H     -3.604958   -1.396939    0.068598
```

M062X
E(SCF)= -285.23009 a.u.
E(SCF+ZPVE)= -285.14536 a.u.

```
11
5a M062X
N     -0.082976   -0.864866    0.000000
C      0.000000    0.538843    0.000000
C      1.031599   -1.655009    0.000000
O      2.169336   -1.234813    0.000000
H      0.787179   -2.730601    0.000000
C     -1.044158    1.331606    0.000000
C     -2.077675    2.131994    0.000000
H     -0.994298   -1.300112    0.000000
H      1.017719    0.916580    0.000000
H     -2.521526    2.481047    0.929441
H     -2.521526    2.481047   -0.929441
```

WB97
E(SCF)= -285.30212 a.u.
E(SCF+ZPVE)= -285.21693 a.u.

```
11
5a WB97
N      0.565085    0.378763   -0.441890
C     -0.666157    0.938475    0.080598
C      1.585026   -0.063445    0.345998
O      2.574820   -0.638477   -0.067984
H      1.434218    0.171931    1.419133
C     -1.799512   -0.006423    0.055295
C     -2.725953   -0.778827    0.020307
H      0.610678    0.116928   -1.418818
H     -0.932999    1.840429   -0.483464
```

```
H     -0.481511    1.256645    1.114313
H     -3.544958   -1.468136   -0.007248
```

WB97(DMSO)
E(SCF)= -285.31564 a.u.
E(SCF+ZPVE)= -285.23033 a.u.

```
11
5a WB97 DMSO
N      0.558168    0.325285   -0.435900
C     -0.663009    0.928334    0.072489
C      1.584614   -0.060939    0.347949
O      2.605385   -0.609282   -0.065878
H      1.434942    0.169491    1.418040
C     -1.810586    0.000151    0.052622
C     -2.750993   -0.757640    0.026464
H      0.623668    0.116963   -1.425600
H     -0.903213    1.820087   -0.516652
H     -0.480801    1.262700    1.100244
H     -3.585013   -1.431420    0.005154
```

WB97X
E(SCF)= -285.27925 a.u.
E(SCF+ZPVE)= -285.19414 a.u.

```
11
5a WB97X
N      0.564108    0.372168   -0.440333
C     -0.666155    0.933350    0.079152
C      1.585610   -0.063171    0.345390
O      2.575985   -0.632966   -0.067606
H      1.435061    0.172116    1.416554
C     -1.799061   -0.006088    0.055209
C     -2.727665   -0.773586    0.021060
H      0.611254    0.113627   -1.416893
H     -0.929845    1.833550   -0.486112
H     -0.482037    1.254440    1.110241
H     -3.547449   -1.458211   -0.005481
```

WB97XD
E(SCF)= -285.25765 **a.u.**
E(SCF+ZPVE)= -285.17297 **a.u.**

```
11
5a WB97XD
N      0.565981    0.386905   -0.436211
C     -0.669355    0.936834    0.085915
C      1.581092   -0.070961    0.346021
O      2.571573   -0.635652   -0.072627
H      1.424240    0.141926    1.420266
C     -1.795193   -0.007304    0.054898
C     -2.719303   -0.781202    0.014258
H      0.615591    0.148252   -1.417676
H     -0.935536    1.838123   -0.475700
H     -0.489312    1.254973    1.118260
H     -3.532880   -1.470600   -0.017219
```

B3LYP
E(SCF)= -285.35967 a.u.
E(SCF+ZPVE)= -285.27615 a.u.

```
11
5a B3LYP
N      0.559239    0.324137   -0.446144
C     -0.672935    0.920125    0.055057
C      1.611435   -0.038447    0.343020
O      2.607538   -0.620307   -0.053356
H      1.479921    0.269525    1.398433
C     -1.817663   -0.000410    0.058124
C     -2.763073   -0.754284    0.042182
H      0.590158    0.005948   -1.407798
H     -0.920129    1.808253   -0.539258
```

H	-0.479639	1.273497	1.074480
H	-3.591864	-1.425631	0.033703

5b

CBS-QB3
E(0 K)= -284.88908 **a.u.**

11
5b CBS-QB3

N	0.000000	0.612568	0.000000
C	-0.324081	-0.753113	0.000000
C	-0.922316	1.626758	0.000000
O	-0.645755	2.803295	0.000000
H	-1.960369	1.243585	0.000000
C	0.538216	-1.736953	0.000000
C	1.381604	-2.729696	0.000000
H	0.973311	0.888975	0.000000
H	-1.390941	-0.956878	0.000000
H	1.751747	-3.165999	0.925149
H	1.751747	-3.165999	-0.925149

M05
E(SCF)= -285.18696 **a.u.**
E(SCF+ZPVE)= -285.10332 **a.u.**

11
5b M05

N	0.000000	0.606947	0.000000
C	-0.354951	-0.747057	0.000000
C	-0.885954	1.647889	0.000000
O	-0.564961	2.818520	0.000000
H	-1.938452	1.306637	0.000000
C	0.495717	-1.749157	0.000000
C	1.331354	-2.756977	0.000000
H	0.980148	0.854423	0.000000
H	-1.425834	-0.935599	0.000000
H	1.693414	-3.195221	0.928356
H	1.693414	-3.195221	-0.928356

M052X
E(SCF)= -285.32308 a.u.
E(SCF+ZPVE)= -285.23794 a.u.

11
5b M052X

N	0.000000	0.610831	0.000000
C	-0.336669	-0.750741	0.000000
C	-0.920029	1.615032	0.000000
O	-0.637425	2.795476	0.000000
H	-1.957985	1.247661	0.000000
C	0.531588	-1.732336	0.000000
C	1.387741	-2.719583	0.000000
H	0.971930	0.883705	0.000000
H	-1.401558	-0.956139	0.000000
H	1.755614	-3.144545	0.928334
H	1.755614	-3.144545	-0.928334

M06L
E(SCF)= -285.33027 a.u.
E(SCF+ZPVE)= -285.24712 a.u.

11
5b M06L

N	0.000000	0.614816	0.000000

```
C     -0.315679   -0.748039    0.000000
C     -0.934482    1.612690    0.000000
O     -0.674930    2.799802    0.000000
H     -1.967903    1.207165    0.000000
C      0.553173   -1.731181    0.000000
C      1.402191   -2.722814    0.000000
H      0.971481    0.896435    0.000000
H     -1.385304   -0.950578    0.000000
H      1.774972   -3.159544    0.925583
H      1.774972   -3.159544   -0.925583
```

M06HF
E(SCF)= -285.26900 a.u.
E(SCF+ZPVE)= -285.18310 a.u.

```
11
5b M06HF
N      0.000000    0.607065    0.000000
C     -0.354682   -0.752326    0.000000
C     -0.904519    1.624929    0.000000
O     -0.598031    2.794948    0.000000
H     -1.947898    1.288072    0.000000
C      0.509804   -1.736276    0.000000
C      1.367422   -2.723604    0.000000
H      0.979533    0.865907    0.000000
H     -1.419886   -0.951429    0.000000
H      1.732177   -3.143963    0.930340
H      1.732177   -3.143963   -0.930340
```

M06
E(SCF)= -285.18069 a.u.
E(SCF+ZPVE)= -285.09766 a.u.

```
11
5b M06
N      0.000000    0.610640    0.000000
C     -0.332679   -0.750358    0.000000
C     -0.918637    1.621081    0.000000
O     -0.638482    2.799891    0.000000
H     -1.961393    1.239400    0.000000
C      0.531436   -1.735482    0.000000
C      1.383536   -2.723993    0.000000
H      0.976801    0.884337    0.000000
H     -1.404618   -0.949897    0.000000
H      1.757560   -3.157466    0.927861
H      1.757560   -3.157466   -0.927861
```

M062X
E(SCF)= -285.23927 a.u.
E(SCF+ZPVE)=-285.15491 a.u.

```
11
5b M062X
N      0.000000    0.610773    0.000000
C     -0.335849   -0.751205    0.000000
C     -0.919809    1.619842    0.000000
O     -0.636893    2.797906    0.000000
H     -1.960885    1.249186    0.000000
C      0.531249   -1.734599    0.000000
C      1.386403   -2.722950    0.000000
H      0.975858    0.882259    0.000000
H     -1.403664   -0.955532    0.000000
H      1.755936   -3.150549    0.929197
H      1.755936   -3.150549   -0.929197
```

WB97
E(SCF)= -285.31011 a.u.
E(SCF+ZPVE)= -285.22538 a.u.

```
11
5b Wb97
N      0.000000    0.611761    0.000000
```

```
C     -0.338648   -0.754498    0.000000
C     -0.920253    1.620650    0.000000
O     -0.635371    2.803414    0.000000
H     -1.965288    1.252847    0.000000
C      0.530338   -1.737485    0.000000
C      1.389145   -2.725492    0.000000
H      0.975601    0.883365    0.000000
H     -1.408572   -0.960598    0.000000
H      1.758868   -3.152151    0.932113
H      1.758868   -3.152151   -0.932113
```

WB97(DMSO)
E(SCF)= -285.32083 a.u.
E(SCF+ZPVE)= -285.23603 a.u.

```
11
5b WB97 DMSO
N      0.000000    0.612858    0.000000
C     -0.348922   -0.755495    0.000000
C     -0.912303    1.613185    0.000000
O     -0.621459    2.806493    0.000000
H     -1.957867    1.258430    0.000000
C      0.523537   -1.735696    0.000000
C      1.381855   -2.724525    0.000000
H      0.980193    0.872884    0.000000
H     -1.419359   -0.956693    0.000000
H      1.751855   -3.150692    0.932291
H      1.751855   -3.150692   -0.932291
```

WB97X
E(SCF)= -285.28873 **a.u.**
E(SCF+ZPVE)= -285.20400 **a.u.**

```
11
5b WB97X
N      0.000000    0.610546    0.000000
C     -0.339439   -0.752799    0.000000
C     -0.915476    1.620960    0.000000
O     -0.627615    2.800670    0.000000
H     -1.959862    1.257601    0.000000
C      0.526178   -1.736025    0.000000
C      1.381039   -2.724551    0.000000
H      0.975215    0.879474    0.000000
H     -1.407786   -0.956799    0.000000
H      1.749772   -3.152487    0.929952
H      1.749772   -3.152487   -0.929952
```

WB97XD
E(SCF)= -285.26879 a.u.
E(SCF+ZPVE)= -285.18441 a.u.

```
11
5b WB97XD
N     -0.080925   -0.859448    0.000000
C      0.000000    0.544545    0.000000
C      1.026775   -1.657927    0.000000
O      2.171703   -1.251595    0.000000
H      0.770654   -2.731204    0.000000
C     -1.043589    1.336057    0.000000
C     -2.076365    2.136540    0.000000
H     -0.992242   -1.292858    0.000000
H      1.015943    0.926074    0.000000
H     -2.521216    2.485800    0.929169
H     -2.521216    2.485800   -0.929169
```

B3LYP
E(SCF)= -285.37407 a.u.
E(SCF+ZPVE)= -285.29077 a.u.

```
11
5b b3lyp
N      0.000000    0.610826    0.000000
```

```
C     -0.343535   -0.753415    0.000000
C     -0.906141    1.636589    0.000000
O     -0.603727    2.818040    0.000000
H     -1.953922    1.283232    0.000000
C      0.516012   -1.746730    0.000000
C      1.360343   -2.746719    0.000000
H      0.979405    0.872687    0.000000
H     -1.414027   -0.946262    0.000000
H      1.729143   -3.184059    0.927120
H      1.729143   -3.184059   -0.927120
```

5c

CBS-QB3
E(0 K)= -284.88748 **a.u.**

```
11
5c CBS-QB3
N     -0.805135    0.553084    0.000047
C      0.521727    0.298223    0.000260
C     -1.793526   -0.418193    0.000106
O     -2.971699   -0.165744   -0.000227
H     -1.367704   -1.435844    0.000504
C      1.700283    0.056495    0.000535
C      3.130265   -0.226278    0.000115
H     -1.131179    1.512621   -0.000305
H      3.414516   -0.851711   -0.851217
H      3.441071   -0.736880    0.916338
H      3.700334    0.704698   -0.069930
```

M05
E(SCF)= -285.18651 a.u.
E(SCF+ZPVE)= -285.10276 a.u.

```
11
5c M05
N     -0.808737    0.547745    0.000135
C      0.518367    0.293589    0.000532
C     -1.795952   -0.418317    0.000603
O     -2.978344   -0.160146   -0.000641
H     -1.378055   -1.439661    0.000542
C      1.709187    0.054984    0.000822
C      3.139216   -0.222610    0.000354
H     -1.131610    1.507379   -0.000475
H      3.408760   -0.906111   -0.809553
H      3.463230   -0.664677    0.946640
H      3.700685    0.704153   -0.146840
```

M052X
E(SCF)= -285.31857 a.u.
E(SCF+ZPVE)= -285.23348 a.u.

```
11
5c M052X
N      0.815068   -0.562228    0.000006
C     -0.515404   -0.300016   -0.000038
C      1.780734    0.417746   -0.000007
O      2.966034    0.175715    0.000014
H      1.355683    1.430532   -0.000027
C     -1.699029   -0.062854    0.000037
C     -3.134003    0.226466    0.000048
H      1.141183   -1.518688    0.000013
H     -3.616803   -0.194255   -0.882671
H     -3.293086    1.305613   -0.007713
H     -3.614515   -0.181382    0.890005
```

M06L
E(SCF)= -285.32663 a.u.
E(SCF+ZPVE)= -285.24342 a.u.

```
11
5c M06L
```

```
N     -0.806870    0.554528    0.000134
C      0.517906    0.299928    0.000625
C     -1.788184   -0.417721    0.000569
O     -2.974470   -0.168357   -0.000677
H     -1.355920   -1.436113    0.000806
C      1.705841    0.057327    0.000902
C      3.126367   -0.226626    0.000274
H     -1.130036    1.514803   -0.000557
H      3.400710   -0.910943   -0.808820
H      3.456282   -0.673165    0.943830
H      3.701223    0.693135   -0.144999
```

M06HF
E(SCF)= -285.26524 **a.u.**
E(SCF+ZPVE)= -285.17926 **a.u.**

```
11
5c M06HF
N      0.820327   -0.564447   -0.000007
C     -0.514148   -0.299879   -0.000003
C      1.781248    0.419512   -0.000005
O      2.961657    0.177042    0.000001
H      1.364124    1.431276    0.000017
C     -1.694531   -0.063219    0.000067
C     -3.144149    0.227896    0.000034
H      1.145131   -1.524509   -0.000002
H     -3.613206   -0.200925   -0.884450
H     -3.291828    1.307511   -0.009894
H     -3.610291   -0.184423    0.893808
```

M06
E(SCF)= -285.17993 **a.u.**
E(SCF+ZPVE)= -285.09680 **a.u.**

```
11
5c M06
N     -0.810241    0.559539   -0.000036
C      0.516700    0.300091    0.000600
C     -1.785377   -0.416755    0.000164
O     -2.968759   -0.173417   -0.000374
H     -1.350356   -1.435060    0.000878
C      1.701807    0.056979    0.001112
C      3.127041   -0.227592    0.000331
H     -1.141057    1.519716   -0.000491
H      3.398690   -0.912624   -0.810563
H      3.454667   -0.673354    0.946215
H      3.698790    0.695541   -0.146033
```

M062X
E(SCF)= -285.23721 **a.u.**
E(SCF+ZPVE)= -285.15276 **a.u.**

```
11
5c M062X
N      0.814538   -0.562351    0.000010
C     -0.516606   -0.301523   -0.000052
C      1.784296    0.418088   -0.000009
O      2.967519    0.175844    0.000022
H      1.356424    1.433963   -0.000053
C     -1.700407   -0.063252    0.000012
C     -3.134458    0.227173    0.000064
H      1.140092   -1.522409    0.000028
H     -3.621096   -0.192883   -0.883751
H     -3.295508    1.308292   -0.007606
H     -3.618782   -0.180167    0.891044
```

WB97
E(SCF)= -285.30844 a.u.
E(SCF+ZPVE)= -285.22364 a.u.

```
11
5c WB97
```

```
N      0.818457   -0.565619   -0.000003
C     -0.518640   -0.302517   -0.000030
C      1.784446    0.417679   -0.000010
O      2.973588    0.178794    0.000019
H      1.355617    1.436351   -0.000040
C     -1.702246   -0.063540    0.000040
C     -3.140741    0.228233    0.000051
H      1.143614   -1.525113    0.000013
H     -3.627711   -0.193430   -0.885508
H     -3.301114    1.311536   -0.008357
H     -3.625223   -0.179493    0.893457
```

WB97(DMSO)
E(SCF)= -285.31781 **a.u.**
E(SCF+ZPVE)= -285.23313 **a.u.**

```
11
5c WB97 DMSO
N      0.822349   -0.565674   -0.000009
C     -0.516791   -0.300007    0.000135
C      1.775967    0.415096    0.000028
O      2.975164    0.180866   -0.000092
H      1.353523    1.433847    0.000219
C     -1.701362   -0.063441    0.000326
C     -3.140880    0.227609   -0.000015
H      1.138725   -1.529769   -0.000192
H     -3.629223   -0.213639   -0.874787
H     -3.301723    1.310124   -0.030238
H     -3.620660   -0.163314    0.902957
```

WB97X
E(SCF)= -285.28581 a.u.
E(SCF+ZPVE)= -285.20112 a.u.

```
11
5c WB97X
N     -0.815253    0.560982   -0.000011
C      0.517847    0.297448    0.000617
C     -1.784266   -0.416846    0.000211
O     -2.970073   -0.173529   -0.000443
H     -1.360559   -1.435364    0.000970
C      1.699639    0.056907    0.001182
C      3.135735   -0.227275    0.000196
H     -1.138196    1.520231   -0.000609
H      3.398833   -0.912977   -0.810185
H      3.454570   -0.670549    0.947793
H      3.698977    0.698609   -0.147578
```

WB97XD
E(SCF)= -285.264109 a.u.
E(SCF+ZPVE)= -285.17985 a.u.

```
11
5c WB97XD
N     -0.812863    0.558851    0.000001
C      0.517617    0.297162    0.000617
C     -1.785433   -0.417124    0.000219
O     -2.970432   -0.171415   -0.000449
H     -1.362950   -1.435304    0.000972
C      1.700490    0.056748    0.001151
C      3.134101   -0.226836    0.000210
H     -1.134241    1.518968   -0.000592
H      3.398182   -0.912101   -0.810306
H      3.453795   -0.670658    0.947341
H      3.698056    0.698766   -0.147006
```

6

6a

CBS-QB3
E(0 K)=-324.10551 a.u.

14
6a CBS-QB3

C	0.601571	1.543039	0.019159
N	0.470162	0.122039	0.306461
C	1.458541	-0.751564	-0.054374
O	2.506568	-0.450814	-0.583137
H	1.198820	-1.795649	0.205250
C	-0.771300	-0.357447	0.896719
C	-1.931093	-0.253005	0.003314
C	-2.881647	-0.158993	-0.724144
H	1.588372	1.702595	-0.410600
H	0.503043	2.129751	0.938257
H	-0.163608	1.868397	-0.691611
H	-0.976484	0.200125	1.819067
H	-0.629474	-1.402830	1.187200
H	-3.720771	-0.082327	-1.371739

WB97
E(SCF)= -324.60816 a.u.
E(SCF+ZPVE)= -324.49447 a.u.

14
6a WB97

C	0.562566	1.531497	0.054800
N	0.473231	0.107504	0.324908
C	1.464903	-0.740509	-0.068653
O	2.505778	-0.403160	-0.607957
H	1.236806	-1.799190	0.165554
C	-0.757144	-0.399406	0.900508
C	-1.917702	-0.260486	-0.002337
C	-2.865404	-0.137622	-0.739296
H	1.553004	1.736575	-0.357611
H	0.425372	2.100500	0.983273
H	-0.205824	1.834805	-0.667914
H	-0.968644	0.131233	1.839745
H	-0.617973	-1.458051	1.152127
H	-3.704896	-0.033960	-1.396004

WB97(DMSO)
E(SCF)= -324.61998 a.u.
E(SCF+ZPVE)= -324.50632 a.u.

14
6a WB97-DMSO

C	0.557589	1.534056	0.068198
N	0.470763	0.100517	0.298715
C	1.463193	-0.737432	-0.070267
O	2.521005	-0.394639	-0.600899
H	1.244746	-1.795445	0.158009
C	-0.754353	-0.413389	0.891668
C	-1.919834	-0.262717	-0.002772
C	-2.874808	-0.130965	-0.730736
H	1.548829	1.763458	-0.326908
H	0.404161	2.072013	1.011059
H	-0.205118	1.851155	-0.653382
H	-0.950150	0.114509	1.834070
H	-0.614519	-1.472565	1.135064
H	-3.722054	-0.016953	-1.378266

6b

CBS-QB3
E(0 K)= -324.11330 a.u.

14
6b CBS-QB3

C	-1.448497	0.351211	0.000000
N	0.000000	0.510557	0.000000
C	0.554072	1.769490	0.000000
O	-0.069247	2.807172	0.000000
H	1.658510	1.732328	0.000000
C	0.829345	-0.622831	0.000000
C	0.425235	-1.868763	0.000000
C	0.072378	-3.123015	0.000000
H	-1.889433	1.345212	0.000000
H	-1.768036	-0.199927	0.888130
H	-1.768036	-0.199927	-0.888130
H	1.892154	-0.398235	0.000000
H	-0.083191	-3.673639	-0.925002
H	-0.083191	-3.673639	0.925002

WB97
E(SCF)= -324.61703 a.u.
E(SCF+ZPVE)= -324.50361 a.u.

14
6b WB97

C	-1.437385	0.306207	0.000000
N	0.000000	0.513940	0.000000
C	0.523088	1.779347	0.000000
O	-0.132190	2.806034	0.000000
H	1.630146	1.780788	0.000000
C	0.856924	-0.602262	0.000000
C	0.457086	-1.854310	0.000000
C	0.100610	-3.114235	0.000000
H	-1.919491	1.285869	0.000000
H	-1.734474	-0.258596	0.891837
H	-1.734474	-0.258596	-0.891837
H	1.921223	-0.368091	0.000000
H	-0.053675	-3.657853	-0.931790
H	-0.053675	-3.657853	0.931790

WB97(DMSO)
E(SCF)= -324.62607 a.u.
E(SCF+ZPVE)= 324.51282 a.u.

14
6b WB97-DMSO

C	-1.437545	0.295181	0.000000
N	0.000000	0.517542	0.000000
C	0.521906	1.771567	0.000000
O	-0.137501	2.808361	0.000000
H	1.625434	1.779834	0.000000
C	0.865627	-0.598782	0.000000
C	0.460145	-1.848872	0.000000
C	0.098724	-3.107632	0.000000
H	-1.936112	1.265508	0.000000
H	-1.726652	-0.271654	0.892231
H	-1.726652	-0.271654	-0.892231
H	1.928523	-0.360907	0.000000
H	-0.058836	-3.649788	-0.932084
H	-0.058836	-3.649788	0.932084

6c

CBS-QB3
E(0 K)= -324.10954 **a.u.**

14
6c CBS-QB3

C	1.201652	1.559504	-0.000064
N	0.637568	0.203176	0.000123
C	1.480515	-0.899776	0.000068
O	2.686318	-0.837240	-0.000194
H	0.908774	-1.842239	0.000272
C	-0.705163	0.041207	0.000284
C	-1.902384	-0.083505	0.000437
C	-3.351979	-0.238282	0.000027

```
H      0.876613    2.100330   -0.890959
H      0.876475    2.100592    0.890619
H      2.285018    1.459771    0.000014
H     -3.838808    0.739812   -0.054705
H     -3.692360   -0.824408   -0.858778
H     -3.705074   -0.733057    0.909718
```

WB97
E(SCF)= -324.61324 a.u.
E(SCF+ZPVE)= -324.49991 a.u.

```
14
6c WB97
C      1.200454    1.559335   -0.000074
N      0.649735    0.205392    0.000154
C      1.478813   -0.897828    0.000057
O      2.692480   -0.838446   -0.000194
H      0.911240   -1.845635    0.000251
C     -0.703663    0.038505    0.000282
C     -1.905588   -0.084748    0.000406
C     -3.365547   -0.238263    0.000003
H      0.869066    2.097029   -0.894621
H      0.869068    2.097298    0.894311
H      2.289349    1.472375   -0.000073
H     -3.843802    0.745757   -0.049171
H     -3.699244   -0.820543   -0.865403
H     -3.710479   -0.738455    0.911134
```

WB97(DMSO)
E(SCF)= -324.62116 a.u.
E(SCF+ZPVE)= -324.50800 a.u.

```
14
6c WB97-DMSO
C      1.192422    1.565169   -0.000084
N      0.654790    0.202735    0.000171
C      1.474694   -0.894470    0.000056
O      2.697111   -0.839166   -0.000202
H      0.913940   -1.843864    0.000233
C     -0.701162    0.032197    0.000309
C     -1.903820   -0.087752    0.000399
C     -3.364875   -0.238323   -0.000033
H      0.853718    2.096229   -0.894849
H      0.853951    2.096455    0.894635
H      2.281696    1.496754   -0.000214
H     -3.840562    0.746623   -0.048334
H     -3.697963   -0.819895   -0.865619
H     -3.708742   -0.739043    0.910693
```

7a

CBS-QB3
E(0 K)=-438.48928 a.u.

```
18
7a CBS-QB3
C     -0.854145    2.015356   -0.057051
N     -0.301276    0.732933    0.357952
C      0.959534    0.394063   -0.059771
O      1.716013    1.129368   -0.656901
O      1.280186   -0.876667    0.300777
C      2.600441   -1.295701   -0.075802
C     -1.222883   -0.233163    0.951505
C     -2.159012   -0.825217   -0.012998
C     -2.926939   -1.301518   -0.804135
H     -0.043944    2.634733   -0.432308
H     -1.325249    2.510078    0.797648
H     -1.603220    1.886816   -0.845972
H      2.681871   -2.327482    0.260369
H      3.355834   -0.674887    0.408496
H      2.729779   -1.234332   -1.157119
H     -0.643004   -1.020175    1.430441
H     -1.792322    0.278083    1.736059
H     -3.602383   -1.727900   -1.504790
```

WB97
E(SCF)= -439.14555 a.u.
E(SCF+ZPVE)=   -438.99792 a.u.

```
18
7a WB97
C     -0.893352    1.996199   -0.097713
N     -0.307061    0.764604    0.406774
C      0.933063    0.402458   -0.047982
O      1.670321    1.118487   -0.705153
O      1.270425   -0.848415    0.345069
C      2.575249   -1.270935   -0.071095
C     -1.223206   -0.202660    0.990643
C     -2.091130   -0.853804   -0.013239
C     -2.800905   -1.373424   -0.840010
H     -0.091846    2.654150   -0.437258
H     -1.453051    2.486416    0.707681
H     -1.575324    1.795221   -0.936283
H      2.683242   -2.289207    0.307655
H      3.341879   -0.615659    0.353687
H      2.650469   -1.253148   -1.162454
H     -0.651777   -0.961866    1.529818
H     -1.852149    0.323987    1.720155
H     -3.426304   -1.839696   -1.573376
```

WB97(DMSO)
E(SCF)= -439.15508 a.u.
E(SCF+ZPVE)= -439.007881a .u.

```
18
7a WB97(DMSO)
C     -0.881742    2.041836   -0.139454
N     -0.316010    0.807039    0.386928
C      0.913100    0.410234   -0.049126
O      1.676404    1.106937   -0.711898
O      1.217356   -0.840645    0.351686
C      2.522240   -1.309698   -0.026771
C     -1.248228   -0.134080    0.994241
C     -2.039175   -0.880166   -0.007965
C     -2.681796   -1.482099   -0.835756
H     -0.071964    2.718682   -0.415105
H     -1.496786    2.510951    0.635236
H     -1.505534    1.847751   -1.023266
H      2.589659   -2.323473    0.370594
H      3.296645   -0.673953    0.411981
```

```
H      2.621386   -1.317298   -1.115896
H     -0.700195   -0.835616    1.627511
H     -1.926163    0.435241    1.640543
H     -3.251454   -2.018050   -1.569416
```

7b

CBS-QB3
E(0 K)=-438.49664 a.u.

```
18
7b CBS-QB3
C      0.861287    1.768868   -0.000146
N      0.247018    0.446106   -0.000405
C     -1.128825    0.359184    0.000003
O     -1.878546    1.310780    0.000408
O     -1.550425   -0.928679   -0.000247
C     -2.977188   -1.097302    0.000116
C      1.081164   -0.688097   -0.000105
C      2.391861   -0.654718   -0.000399
C      3.694722   -0.685403    0.000439
H      0.069848    2.512314   -0.000156
H      1.488093    1.891998   -0.887567
H      1.487951    1.891762    0.887380
H     -3.138901   -2.173207   -0.005888
H     -3.418808   -0.638158   -0.885283
H     -3.417025   -0.648680    0.891859
H      0.557337   -1.633896    0.000045
H      4.267353   -0.698294    0.925371
H      4.268677   -0.698579   -0.923658
```

WB97
E(SCF)=-439.15402 a.u.
E(SCF+ZPVE)= -439.00672 a.u.

```
18
7b WB97
C      0.097037    1.949855    0.000000
N      0.000000    0.499344    0.000000
C      1.153055   -0.247062    0.000000
O      2.278803    0.220759    0.000000
O      0.900218   -1.574642    0.000000
C      2.070916   -2.403160    0.000000
C     -1.282661   -0.085758    0.000000
C     -2.402629    0.604005    0.000000
C     -3.545252    1.244325    0.000000
H      1.152050    2.226220    0.000000
H     -0.396338    2.356662    0.891273
H     -0.396338    2.356662   -0.891273
H      1.697286   -3.428922    0.000000
H      2.673766   -2.209615    0.892285
H      2.673766   -2.209615   -0.892285
H     -1.301598   -1.170990    0.000000
H     -4.038779    1.521011   -0.931472
H     -4.038779    1.521011    0.931472
```

WB97(DMSO)
E(SCF)= -439.16096 **a.u.**
E(SCF+ZPVE)= -439.01416 **a.u.**

```
18
7b WB97-DMSO
C     -0.886929    1.743620   -0.000003
N     -0.244278    0.437442   -0.000030
C      1.122351    0.355191    0.000000
O      1.872426    1.324956    0.000018
O      1.559979   -0.916872    0.000006
C      2.989250   -1.077957    0.000006
C     -1.068988   -0.709687   -0.000027
C     -2.383795   -0.662664   -0.000009
C     -3.693923   -0.670527    0.000027
```

H	-0.116722	2.514688	-0.000027
H	-1.515857	1.848870	0.891851
H	-1.515920	1.848870	-0.891813
H	3.155648	-2.156007	-0.000160
H	3.421556	-0.622673	0.895403
H	3.421590	-0.622384	-0.895226
H	-0.548466	-1.662207	-0.000049
H	-4.259488	-0.671876	-0.931668
H	-4.259435	-0.671902	0.931754

CBS-QB3
E(0 K)=-438.49164 a.u.

18
7c CBS-QB3

C	0.025697	2.344351	0.000002
N	0.017145	0.875457	-0.000015
C	-1.204405	0.214213	-0.000006
O	-2.277533	0.773046	0.000003
O	-1.032234	-1.119155	-0.000013
C	-2.246495	-1.887125	0.000007
C	1.203380	0.225813	0.000028
C	2.295353	-0.279143	0.000008
C	3.600068	-0.929398	0.000013
H	-1.006973	2.683359	0.000183
H	0.538566	2.713911	-0.890485
H	0.538879	2.713872	0.890322
H	-1.926843	-2.926806	-0.000059
H	-2.839897	-1.668121	-0.888887
H	-2.839808	-1.668212	0.888984
H	4.183916	-0.664153	0.886780
H	4.186660	-0.658326	-0.883172
H	3.482027	-2.017124	-0.003795

WB97
E(SCF)= -439.14847 a.u.
E(SCF+ZPVE)= -439.00134 a.u.

18
7c WB97

C	-0.003948	2.342390	0.000000
N	0.000000	0.879995	0.000000
C	1.204170	0.201313	0.000000
O	2.292564	0.747098	0.000000
O	1.020375	-1.127970	0.000000
C	2.231077	-1.895289	0.000000
C	-1.201284	0.235536	0.000000
C	-2.300814	-0.263357	0.000000
C	-3.621774	-0.903575	0.000000
H	1.033277	2.682502	0.000000
H	-0.517739	2.712014	0.894081
H	-0.517739	2.712014	-0.894081
H	1.909684	-2.938448	0.000000
H	2.823454	-1.671668	0.892707
H	2.823454	-1.671668	-0.892707
H	-4.197516	-0.623408	-0.888819
H	-4.197516	-0.623408	0.888819
H	-3.507431	-1.993024	0.000000

WB97(DMSO)
E(SCF)= -439.15682 a.u.
E(SCF+ZPVE)= -439.01007 a.u.

18
7c WB97-DMSO

C	0.027928	2.347958	0.000004
N	0.001874	0.882957	-0.000012
C	-1.200843	0.210933	-0.000003
O	-2.290405	0.765955	0.000007
O	-1.029412	-1.116708	-0.000007
C	-2.242087	-1.892839	0.000004
C	1.197914	0.225758	-0.000003

C	2.293810	-0.283950	0.000005
C	3.615086	-0.926666	0.000006
H	-1.001599	2.708068	-0.000111
H	0.545787	2.708224	-0.894496
H	0.545589	2.708219	0.894621
H	-1.915662	-2.933457	0.000003
H	-2.829877	-1.673760	-0.895737
H	-2.829864	-1.673757	0.895752
H	4.189046	-0.645318	0.888912
H	4.189528	-0.644270	-0.888257
H	3.501631	-2.015784	-0.000675

8

8a

CBS-QB3
E(0K)=-474.15830 a.u.

```
15
8a CBS-QB3
C      0.471680   -2.105251    0.666243
C      0.471680   -2.105251   -0.666243
C      0.403633   -0.684744   -1.152926
N      0.358780    0.107277    0.000000
C      0.403633   -0.684744    1.152926
O      0.403633   -0.279799    2.288141
O      0.403633   -0.279799   -2.288141
C      0.252014    1.561445    0.000000
C     -1.130033    2.045108    0.000000
C     -2.255747    2.460593    0.000000
H      0.518189   -2.937709    1.352961
H      0.518189   -2.937709   -1.352961
H      0.773797    1.927742   -0.887326
H      0.773797    1.927742    0.887326
H     -3.254711    2.822848    0.000000
```

WB97
E(SCF)=-474.82325 a.u.
E(SCF+ZPVE)= -474.71558 a.u.

```
15
8a WB97
C     -0.389558    2.110025    0.666351
C     -0.389558    2.110025   -0.666351
C     -0.389650    0.682287   -1.148575
N     -0.405548   -0.108814    0.000000
C     -0.389650    0.682287    1.148575
O     -0.389650    0.271555    2.287300
O     -0.389650    0.271555   -2.287300
C     -0.322455   -1.556965    0.000000
C      1.067307   -2.046501    0.000000
C      2.203021   -2.452092    0.000000
H     -0.392224    2.946075    1.357589
H     -0.392224    2.946075   -1.357589
H     -0.845479   -1.919580   -0.891747
H     -0.845479   -1.919580    0.891747
H      3.211894   -2.810568    0.000000
```

WB97(DMSO)
E(SCF)= -474.83574 **a.u.**
E(SCF+ZPVE)=   -474.72826 **a.u.**

```
15
8a WB97 DMSO
C     -0.314228    2.132243    0.666329
C     -0.314228    2.132243   -0.666329
C     -0.353937    0.706150   -1.143613
N     -0.394831   -0.086506    0.000000
C     -0.353937    0.706150    1.143613
O     -0.353937    0.288081    2.284273
O     -0.353937    0.288081   -2.284273
C     -0.398240   -1.537361    0.000000
C      0.963084   -2.104576    0.000000
C      2.076908   -2.570846    0.000000
H     -0.290800    2.970484    1.354038
H     -0.290800    2.970484   -1.354038
H     -0.945374   -1.872422   -0.887233
H     -0.945374   -1.872422    0.887233
```

```
H      3.066613   -2.983895    0.000000
```

8b

CBS-QB3
E(0 K)= -474.16033 a.u.

```
15
8b CBS-QB3
C     -2.262306   -0.278537    0.000000
C     -2.199886    1.069392    0.000000
C     -0.754318    1.486658    0.000000
N      0.000000    0.286011    0.000000
C     -0.861504   -0.838541    0.000000
O     -0.516260   -2.004701    0.000000
O     -0.286035    2.611381    0.000000
C      1.411593    0.290446    0.000000
C      2.211708   -0.765043    0.000000
C      3.091865   -1.743807    0.000000
H     -3.142626   -0.936824    0.000000
H     -3.014011    1.807988    0.000000
H      1.812411    1.321762    0.000000
H      3.469837   -2.185930    0.943320
H      3.469837   -2.185930   -0.943320
```

WB97
E(SCF)= -474.826367721 a.u.
E(SCF+ZPVE)= -474.71933 a.u.

```
15
8b WB97 vacuo
C     -2.241840   -0.309088    0.000000
C     -2.206435    1.022199    0.000000
C     -0.768521    1.463482    0.000000
N      0.000000    0.291960    0.000000
C     -0.829249   -0.836063    0.000000
O     -0.465857   -1.988470    0.000000
O     -0.329243    2.591041    0.000000
C      1.414324    0.319799    0.000000
C      2.203244   -0.728583    0.000000
C      3.064676   -1.710845    0.000000
H     -3.095809   -0.978100    0.000000
H     -3.021873    1.737448    0.000000
H      1.811321    1.333198    0.000000
H      3.424982   -2.141123    0.932919
H      3.424982   -2.141123   -0.932919
```

WB97(DMSO)
E(SCF)= -474.83702 **a.u.**
E(SCF+ZPVE)= -474.73022 **a.u.**

```
15
8b WB97 DMSO
C     -2.252678   -0.276696    0.000000
C     -2.195873    1.053839    0.000000
C     -0.753147    1.468068    0.000000
N      0.000000    0.286947    0.000000
C     -0.850454   -0.821599    0.000000
O     -0.498743   -1.982345    0.000000
O     -0.288307    2.588890    0.000000
C      1.414018    0.294302    0.000000
C      2.196090   -0.759974    0.000000
C      3.063852   -1.738522    0.000000
H     -3.119491   -0.928415    0.000000
H     -3.002370    1.778710    0.000000
H      1.826958    1.301226    0.000000
H      3.430227   -2.164511    0.933054
H      3.430227   -2.164511   -0.933054
```

8c

CBS-QB3
E(0 K)= -474.15518 a.u.
CBSQB3

```
15
8c CBS-QB3
C     -0.693087    2.378508    0.000000
C      0.638943    2.394695    0.000000
C      1.157011    0.989684    0.000000
N      0.000000    0.162472    0.000000
C     -1.176740    0.961321    0.000000
O     -2.304714    0.553140    0.000000
O      2.294618    0.609286    0.000000
C      0.015728   -1.187598    0.000000
C      0.028589   -2.388187    0.000000
C      0.038093   -3.844268    0.000000
H     -1.386765    3.206458    0.000000
H      1.312266    3.239278    0.000000
H     -0.985153   -4.230272    0.000000
H      0.544597   -4.238550    0.885563
H      0.544597   -4.238550   -0.885563
```

WB97
E(SCF)= -474.82067 a.u.
E(SCF+ZPVE)= -474.71351 a.u.

```
15
8c WB97
C      2.396797   -0.665641   -0.001157
C      2.396381    0.666661   -0.001029
C      0.976670    1.160958    0.000222
N      0.172951   -0.000156    0.001619
C      0.977390   -1.160817    0.000469
O      0.577243   -2.297506   -0.000181
O      0.575814    2.297414   -0.000125
C     -1.189091   -0.000454    0.002522
C     -2.393899   -0.000850    0.003324
C     -3.859988   -0.000367   -0.001237
H      3.235184   -1.354074   -0.002221
H      3.234329    1.355632   -0.002000
H     -4.237952   -0.846769   -0.583722
H     -4.234709    0.922678   -0.455870
H     -4.257525   -0.072564    1.016243
```

WB97
E(SCF)= -474.83323 a.u.
E(SCF+ZPVE)= -474.72628 a.u.

```
15
8c WB97(DMSO)
C     -0.679853    2.395087    0.000000
C      0.652821    2.403181    0.000000
C      1.151739    0.988884    0.000000
N      0.000000    0.175724    0.000000
C     -1.161502    0.974838    0.000000
O     -2.295061    0.553524    0.000000
O      2.290377    0.581545    0.000000
C      0.007941   -1.186168    0.000000
C      0.014661   -2.392775    0.000000
C      0.019393   -3.860032    0.000000
H     -1.369487    3.231943    0.000000
H      1.332259    3.248324    0.000000
H     -1.008958   -4.235057    0.000000
H      0.526231   -4.246959    0.889468
H      0.526231   -4.246959   -0.889468
```

9

9a

WB97
E(SCF)= -402.04006 a.u.
E(SCF+ZPVE)= -401.88909 a.u.

```
18
9a WB97
C      2.092581    0.041731   -0.606964
C      1.874899   -1.344315    0.012670
C      0.361926   -1.390985    0.287570
N      0.026357    0.012473    0.478988
C      0.932756    0.875442   -0.081462
O      0.803698    2.088743   -0.150144
C     -1.295647    0.450128    0.869906
C     -2.343990   -0.018943   -0.058028
C     -3.196807   -0.420218   -0.812353
H      2.009457    0.025294   -1.701681
H      3.046722    0.511516   -0.352944
H      2.200645   -2.165130   -0.633478
H      2.421941   -1.421223    0.959796
H      0.109244   -1.972738    1.183278
H     -0.202510   -1.808892   -0.561130
H     -1.524110    0.093475    1.883441
H     -1.273148    1.546258    0.887266
H     -3.956631   -0.762862   -1.484349
```

WB97(DMSO)
E(SCF)= -402.05194 a.u.
E(SCF+ZPVE)= -401.90106 a.u.

```
18
9a WB97 DMSO
C      2.071719    0.176595   -0.623204
C      1.972644   -1.244085   -0.053286
C      0.474889   -1.416677    0.246439
N      0.033306   -0.044363    0.481656
C      0.860038    0.891961   -0.052750
O      0.637073    2.105348   -0.078430
C     -1.302354    0.268344    0.943169
C     -2.336974   -0.079267   -0.053190
C     -3.178288   -0.374306   -0.868855
H      1.972539    0.191641   -1.716670
H      2.991822    0.706982   -0.362380
H      2.343629   -2.010006   -0.739707
H      2.543906   -1.314097    0.879281
H      0.282564   -2.034104    1.131387
H     -0.071960   -1.848797   -0.605234
H     -1.500105   -0.269149    1.878640
H     -1.336368    1.342649    1.153617
H     -3.925788   -0.632745   -1.593025
```

9b

WB97
E(SCF)=-402.048360 a.u.
E(SCF+ZPVE)=-401.897791 a.u.

```
18
9b WB97
C     -2.319087   -0.266258    0.168330
C     -1.621784   -1.584565   -0.190238
C     -0.133480   -1.313757    0.093248
N     -0.028016    0.129663   -0.057671
```

C	-1.234983	0.788727	0.008374
O	-1.375921	2.000014	-0.031405
C	1.192310	0.819128	-0.070288
C	2.369668	0.237336	-0.010850
C	3.557792	-0.311418	0.046251
H	-2.649537	-0.240620	1.215142
H	-3.181255	-0.021557	-0.458074
H	-1.997091	-2.441210	0.377648
H	-1.757650	-1.801389	-1.256237
H	0.536950	-1.818209	-0.612980
H	0.162654	-1.609225	1.111667
H	1.083248	1.900827	-0.132873
H	4.090892	-0.601439	-0.858963
H	4.052649	-0.490075	1.000643

WB97(DMSO)

E(SCF)= -402.05815 a.u.
E(SCF+ZPVE)=-401.90781 a.u.

18
9b WB97 DMSO

C	-2.316579	-0.269143	0.166139
C	-1.618066	-1.587399	-0.189705
C	-0.131454	-1.314078	0.091986
N	-0.030500	0.135992	-0.052650
C	-1.233008	0.780725	0.009455
O	-1.380144	2.002443	-0.030645
C	1.192677	0.825302	-0.072471
C	2.368273	0.239064	-0.011719
C	3.554373	-0.313768	0.046814
H	-2.647664	-0.244415	1.212772
H	-3.177530	-0.028761	-0.463836
H	-1.990699	-2.439024	0.385656
H	-1.755686	-1.808090	-1.254060
H	0.538034	-1.809739	-0.619976
H	0.166507	-1.606691	1.109091
H	1.093541	1.906942	-0.143402
H	4.082255	-0.615806	-0.857493
H	4.048601	-0.490125	1.001963

9c

WB97
E(SCF)= -402.040714899 a.u.
E(SCF+ZPVE)= -401.890063 a.u

18
9c WB97

C	-2.390507	0.454095	0.140805
C	-2.347179	-1.036366	-0.213985
C	-0.912430	-1.460684	0.138974
N	-0.156616	-0.217202	-0.029125
C	-0.949694	0.923392	0.002329
O	-0.541016	2.064990	-0.049814
C	1.201893	-0.181146	-0.017078
C	2.409257	-0.167101	-0.009515
C	3.875882	-0.120314	-0.002776
H	-2.690311	0.622655	1.183764
H	-3.044968	1.055821	-0.495442
H	-3.092982	-1.631842	0.321269
H	-2.514598	-1.169946	-1.289297
H	-0.515231	-2.234492	-0.526417
H	-0.826955	-1.814634	1.176759
H	4.292387	-0.607311	-0.891059
H	4.282281	-0.613167	0.886958
H	4.211482	0.922156	0.003325

WB97(DMSO)
E(SCF)= -402.05333 a.u.
E(SCF+ZPVE)=-401.90286 a.u

```
18
9c WB97 DMSO
C     -2.389851    0.457495    0.133063
C     -2.349718   -1.035068   -0.215323
C     -0.918149   -1.464156    0.139789
N     -0.159679   -0.213172   -0.020270
C     -0.950408    0.914726    0.004121
O     -0.530154    2.064401   -0.047541
C      1.199751   -0.171231   -0.012725
C      2.408690   -0.155879   -0.010067
C      3.877408   -0.131089   -0.007417
H     -2.694595    0.630056    1.173828
H     -3.039286    1.056060   -0.511338
H     -3.094857   -1.621966    0.328070
H     -2.519665   -1.173355   -1.288932
H     -0.517752   -2.230620   -0.530403
H     -0.831584   -1.815424    1.176420
H      4.280629   -0.560528   -0.930266
H      4.274788   -0.696514    0.841721
H      4.234970    0.900495    0.074478
```

10

10a

WB97
E(SCF)= -594.942254 a.u.
E(SCF+ZPVE)= -594.71663 a.u.

```
27
10a WB97 vacuo
C     -2.945769    1.135751    0.459515
C     -3.302820    0.637392   -0.794959
C     -2.647003   -0.484409   -1.302537
C     -1.634441   -1.098317   -0.562863
C     -1.260950   -0.601594    0.690639
C     -1.933308    0.519019    1.194032
C     -0.101957   -1.210839    1.445530
C      1.200939   -0.422560    1.238453
N      1.559856   -0.287128   -0.170198
C      1.264124    0.953419   -0.869213
C      2.056388    2.093480   -0.368232
C      2.695168    3.028236    0.049879
C      2.136564   -1.336594   -0.811136
O      2.372396   -2.417950   -0.291286
H      2.378284   -1.122619   -1.870934
H     -3.458723    2.005826    0.867089
H     -4.093432    1.117109   -1.369793
H     -2.925576   -0.885409   -2.276222
H     -1.123606   -1.976043   -0.961255
H     -1.657763    0.914078    2.173360
H     -0.317968   -1.236613    2.522178
H      0.065275   -2.244135    1.118261
H      2.023920   -0.935052    1.749797
H      1.115925    0.589613    1.654409
H      1.472839    0.806740   -1.936090
H      0.193587    1.186501   -0.780348
H      3.267468    3.855590    0.416577
```

WB97(DMSO)
E(SCF)= -594.95575 a.u.
E(SCF+ZPVE)= -594.73015 a.u.

```
27
10a WB97 dmso
C      3.418852   -0.383035    0.220955
C      3.298512    0.978829    0.508635
C      2.130824    1.656743    0.155822
C      1.088866    0.976565   -0.479555
C      1.198419   -0.388240   -0.771887
C      2.375787   -1.058567   -0.413700
C      0.053093   -1.146634   -1.406065
C     -0.848482   -1.815602   -0.355627
N     -1.583261   -0.866179    0.476661
C     -2.818504   -0.276374   -0.023427
C     -2.623655    0.981675   -0.773340
C     -2.487001    2.012406   -1.388985
C     -1.093932   -0.448353    1.665198
O     -1.619538    0.411638    2.374621
H     -0.166015   -0.967819    1.962154
H      4.328425   -0.919357    0.488236
H      4.112066    1.508101    1.002527
H      2.028753    2.718898    0.374738
H      0.176534    1.509373   -0.750931
H      2.477217   -2.121557   -0.638204
H     -0.550267   -0.476695   -2.032122
H      0.444302   -1.937737   -2.058680
H     -0.241785   -2.445672    0.306769
H     -1.579475   -2.469551   -0.845860
H     -3.314361   -1.011076   -0.667419
H     -3.473700   -0.079884    0.831954
H     -2.359237    2.926653   -1.934893
```

10b
WB97
E(SCF)= -594.95038 a.u.
E(SCF+ZPVE)= -594.72512 a.u.

27
10b WB97 vacuo

C	-2.417233	0.489611	1.465823
C	-3.555303	0.310042	0.679208
C	-3.487469	-0.492915	-0.459820
C	-2.285659	-1.109226	-0.809349
C	-1.137631	-0.936233	-0.027866
C	-1.218475	-0.130894	1.114169
C	0.179681	-1.547736	-0.445223
C	1.002800	-0.593403	-1.327111
N	1.377301	0.638257	-0.648146
C	2.417085	0.633275	0.304354
C	3.150108	-0.414365	0.608681
C	3.895346	-1.437677	0.945420
C	0.661418	1.796547	-0.828245
O	0.879221	2.844837	-0.246492
H	-0.145534	1.682718	-1.575812
H	-2.460397	1.117413	2.354393
H	-4.491781	0.793239	0.953075
H	-4.372714	-0.641916	-1.076583
H	-2.239173	-1.738878	-1.699533
H	-0.330049	0.014916	1.730650
H	0.770992	-1.821985	0.437612
H	0.006345	-2.469504	-1.016354
H	0.423399	-0.319178	-2.217855
H	1.921813	-1.088864	-1.666535
H	2.577462	1.599032	0.779823
H	3.611910	-2.098290	1.764965
H	4.824842	-1.657355	0.420867

WB97(DMSO)
E(SCF)= -594.96159 a.u.
E(SCF+ZPVE)= -594.73656 a.u.
27
10b WB97 DMSO

C	-2.410108	0.287794	1.559452
C	-3.528072	0.249309	0.724207
C	-3.441255	-0.386066	-0.516618
C	-2.241989	-0.976805	-0.918003
C	-1.114008	-0.944488	-0.088328
C	-1.212763	-0.305388	1.153689
C	0.200248	-1.530729	-0.551064
C	1.021948	-0.523668	-1.371417
N	1.359484	0.682424	-0.620202
C	2.375516	0.642200	0.362440
C	3.124178	-0.408099	0.614897
C	3.877791	-1.439468	0.903034
C	0.637394	1.825294	-0.775571
O	0.815473	2.862048	-0.139551
H	-0.141272	1.738406	-1.552488
H	-2.469410	0.779671	2.529430
H	-4.463354	0.709532	1.039332
H	-4.310098	-0.424024	-1.172373
H	-2.180500	-1.473003	-1.887738
H	-0.341330	-0.273554	1.809835
H	0.789921	-1.867274	0.310903
H	0.024013	-2.408162	-1.186098
H	0.458703	-0.214751	-2.259640
H	1.956055	-0.984429	-1.714920
H	2.501599	1.574558	0.907917
H	3.577815	-2.161293	1.662686
H	4.824417	-1.608355	0.390666

10c
WB97
E(SCF)= -594.94601 a.u.
E(SCF+ZPVE)= -594.72102 a.u.

```
27
10c wb97
C     -3.458776   -0.144455    0.039392
C     -3.196628   -1.205128   -0.828547
C     -1.931086   -1.790913   -0.838815
C     -0.934018   -1.318081    0.015433
C     -1.187096   -0.255947    0.890197
C     -2.459644    0.325014    0.890596
C     -0.090408    0.287030    1.778649
C      0.673157    1.446071    1.125758
N      1.303300    1.046899   -0.136362
C      2.287015    0.103821   -0.120831
C      3.157015   -0.734710   -0.085147
C      4.225726   -1.740931   -0.046841
C      0.837979    1.530873   -1.346055
O     -0.045075    2.356626   -1.455077
H      1.372624    1.087101   -2.205104
H     -4.443884    0.319585    0.052429
H     -3.975973   -1.572857   -1.494202
H     -1.718311   -2.619334   -1.513255
H      0.056165   -1.777208    0.004470
H     -2.669644    1.157014    1.564388
H     -0.512415    0.654305    2.723540
H      0.618720   -0.512675    2.032238
H      1.454499    1.818744    1.800092
H     -0.003539    2.269700    0.875931
H      4.388389   -2.074160    0.983680
H      5.168327   -1.329723   -0.423378
H      3.963131   -2.617653   -0.648402
```

10c
WB97(DMSO)
E(SCF)= -594.95709 a.u.
E(SCF+ZPVE)= -594.73215 a.u.

```
27
10c WB97 DMSO
C     -3.488965   -0.138972    0.096440
C     -3.239044   -1.166946   -0.815695
C     -1.968595   -1.742221   -0.875198
C     -0.954755   -1.289915   -0.027765
C     -1.193826   -0.258493    0.888644
C     -2.472530    0.310075    0.940744
C     -0.080019    0.264499    1.767755
C      0.682178    1.426101    1.118838
N      1.308097    1.031131   -0.150654
C      2.322718    0.115729   -0.126903
C      3.215130   -0.698204   -0.077684
C      4.302922   -1.683523   -0.020827
C      0.831549    1.476257   -1.357782
O     -0.093704    2.266186   -1.483788
H      1.381753    1.050952   -2.213804
H     -4.478690    0.312409    0.152420
H     -4.031736   -1.519682   -1.473830
H     -1.765671   -2.546585   -1.581092
H      0.036168   -1.745044   -0.075567
H     -2.673671    1.112866    1.651628
H     -0.484334    0.626467    2.721359
H      0.627266   -0.542399    2.001657
H      1.470356    1.788789    1.788512
H      0.008063    2.255614    0.886097
H      4.502092   -1.957786    1.020241
H      5.224515   -1.275827   -0.448347
H      4.036257   -2.593497   -0.567807
```

11

11a

WB97
E(SCF)=-746.14764 a.u.
E(SCF+ZPVE)= -745.91894 a.u.

```
29
11a WB97
C      1.128781    0.173631    3.668339
C     -0.262548    0.003403    3.632059
C     -0.935210   -0.176160    2.433289
C     -0.221063   -0.198851    1.215894
C      1.172647    0.000702    1.249017
C      1.829981    0.178640    2.475935
N     -0.873376   -0.413840    0.000000
C     -0.221063   -0.198851   -1.215894
C      1.172647    0.000702   -1.249017
C      1.963372    0.042737    0.000000
C     -0.935210   -0.176160   -2.433289
C     -0.262548    0.003403   -3.632059
C      1.128781    0.173631   -3.668339
C      1.829981    0.178640   -2.475935
O      3.187626    0.139452    0.000000
C     -2.248391   -0.891772    0.000000
C     -3.256573    0.185324    0.000000
C     -4.081128    1.066305    0.000000
H      1.642807    0.313322    4.616805
H     -0.836457    0.024987    4.557589
H     -2.018973   -0.257192    2.445587
H      2.908947    0.321376    2.443022
H     -2.018973   -0.257192   -2.445587
H     -0.836457    0.024987   -4.557589
H      1.642807    0.313322   -4.616805
H      2.908947    0.321376   -2.443022
H     -2.393988   -1.534797    0.874230
H     -2.393988   -1.534797   -0.874230
H     -4.806771    1.853930    0.000000
```

WB97(DMSO)
E(SCF)= -746.16052 a.u.
E(SCF+ZPVE)= -745.93196 a.u.

```
29
11a WB97 DMSO
C      1.125159    0.166062    3.668644
C     -0.265728   -0.031316    3.632052
C     -0.935173   -0.217566    2.434719
C     -0.223599   -0.219428    1.212392
C      1.168471    0.004263    1.245330
C      1.823512    0.187943    2.477301
N     -0.875081   -0.433481    0.000000
C     -0.223599   -0.219428   -1.212392
C      1.168471    0.004263   -1.245330
C      1.951535    0.062882    0.000000
C     -0.935173   -0.217566   -2.434719
C     -0.265728   -0.031316   -3.632052
C      1.125159    0.166062   -3.668644
C      1.823512    0.187943   -2.477301
O      3.182672    0.183976    0.000000
C     -2.265694   -0.880709    0.000000
C     -3.233554    0.232546    0.000000
C     -4.023767    1.145911    0.000000
H      1.636603    0.311060    4.617519
H     -0.838681   -0.028354    4.557940
H     -2.014920   -0.332500    2.454044
H      2.899359    0.351345    2.454247
```

```
H     -2.014920   -0.332500   -2.454044
H     -0.838681   -0.028354   -4.557940
H      1.636603    0.311060   -4.617519
H      2.899359    0.351345   -2.454247
H     -2.429879   -1.521247    0.870891
H     -2.429879   -1.521247   -0.870891
H     -4.723592    1.958681    0.000000
```

11b

WB97
E(SCF)=-746.15154 a.u.
E(SCF+ZPVE)= -745.92416 a.u.

```
29
11b WB97
C      3.792659   -0.832152    0.026824
C      3.636187    0.536723   -0.240699
C      2.379413    1.107821   -0.352675
C      1.223381    0.311541   -0.204384
C      1.377135   -1.061484    0.064257
C      2.662930   -1.614589    0.178630
N     -0.048979    0.876542   -0.317511
C     -1.204567    0.095422   -0.218776
C     -1.111285   -1.281793    0.058497
C      0.200880   -1.940307    0.231241
C     -2.484024    0.664280   -0.398478
C     -3.617465   -0.124397   -0.294977
C     -3.527978   -1.495527   -0.008381
C     -2.277389   -2.058091    0.163870
O      0.306513   -3.137477    0.486660
C     -0.154437    2.262792   -0.668134
C     -0.402346    3.187019    0.228740
C     -0.672619    4.110137    1.112578
H      4.786062   -1.266685    0.114782
H      4.514374    1.169989   -0.360326
H      2.285710    2.172618   -0.546655
H      2.725141   -2.680859    0.390068
H     -2.582347    1.722888   -0.619460
H     -4.593528    0.336944   -0.440123
H     -4.427316   -2.102126    0.072832
H     -2.147299   -3.116700    0.382656
H     -0.015237    2.521427   -1.719785
H     -1.702523    4.378480    1.340701
H      0.124866    4.623674    1.645797
```

WB97(DMSO)

E(SCF)= -746.16338 a.u.
E(SCF+ZPVE)= -745.93627 a.u.

```
29
11b WB97-DMSO
C      3.728081   -1.025372    0.019021
C      3.639040    0.349737   -0.266308
C      2.414031    0.981115   -0.381273
C      1.217014    0.246001   -0.217030
C      1.300974   -1.132582    0.064385
C      2.563026   -1.747680    0.181354
N     -0.020730    0.873537   -0.329469
C     -1.208829    0.154439   -0.223298
C     -1.188182   -1.226389    0.060410
C      0.085030   -1.943275    0.235912
C     -2.458385    0.793464   -0.398663
C     -3.631530    0.069873   -0.287927
C     -3.616215   -1.306984    0.002429
C     -2.399825   -1.936722    0.172252
O      0.130306   -3.151705    0.501629
C     -0.066352    2.269300   -0.677564
C     -0.170276    3.199466    0.240928
C     -0.283896    4.135560    1.144813
H      4.699162   -1.507119    0.109160
```

```
H      4.547407    0.935513   -0.398740
H      2.372557    2.045080   -0.595183
H      2.583553   -2.813203    0.402688
H     -2.498607    1.855698   -0.620026
H     -4.581452    0.583281   -0.428945
H     -4.547639   -1.862066    0.089013
H     -2.339161   -3.000376    0.394991
H     -0.009707    2.515169   -1.738629
H     -1.264405    4.481263    1.467294
H      0.598727    4.585959    1.594982
```

11c

WB97(DMSO)

E(SCF)= -746.15557 a.u.
E(SCF+ZPVE)= -745.92777 a.u.

```
29
11c WB97
C      3.674906   -1.260800    0.000000
C      3.643402    0.142008    0.000000
C      2.441894    0.831829    0.000000
C      1.227948    0.121550    0.000000
C      1.249770   -1.282333    0.000000
C      2.480519   -1.958124    0.000000
N      0.000000    0.811446    0.000000
C     -1.229672    0.124433    0.000000
C     -1.254557   -1.279407    0.000000
C     -0.003309   -2.070185    0.000000
C     -2.442006    0.837404    0.000000
C     -3.645005    0.150200    0.000000
C     -3.679635   -1.252553    0.000000
C     -2.486815   -1.952498    0.000000
O     -0.004711   -3.297094    0.000000
C      0.002149    2.175950    0.000000
C      0.004749    3.383396    0.000000
C      0.012857    4.851491    0.000000
H      4.625549   -1.789729    0.000000
H      4.575110    0.705938    0.000000
H      2.428890    1.918383    0.000000
H      2.448758   -3.046329    0.000000
H     -2.426601    1.923913    0.000000
H     -4.575439    0.716231    0.000000
H     -4.631448   -1.779379    0.000000
H     -2.457472   -3.040767    0.000000
H     -0.488504    5.247047    0.889598
H     -0.488504    5.247047   -0.889598
H      1.044170    5.220104    0.000000
```

WB97(DMSO)

E(SCF)= -746.16548 a.u.
E(SCF+ZPVE)= -745.93804 a.u.

```
29
11c WB97 DMSO
C      3.648747   -1.332184   -0.000092
C      3.642700    0.073209    0.000010
C      2.454374    0.783844    0.000066
C      1.225864    0.095551    0.000020
C      1.220500   -1.310148   -0.000080
C      2.442833   -2.007243   -0.000135
N      0.015235    0.808130    0.000078
C     -1.224615    0.147593    0.000030
C     -1.278567   -1.257127   -0.000071
C     -0.045631   -2.066999   -0.000126
C     -2.422887    0.887155    0.000086
C     -3.640185    0.227327    0.000040
C     -3.705603   -1.176573   -0.000061
C     -2.529314   -1.901958   -0.000114
O     -0.071769   -3.301422   -0.000235
```

C	0.045417	2.175171	0.000195
C	0.075511	3.382477	0.000276
C	0.118475	4.850021	0.000151
H	4.589713	-1.877784	-0.000135
H	4.584185	0.619965	0.000046
H	2.463778	1.870471	0.000144
H	2.402784	-3.094777	-0.000212
H	-2.385935	1.973198	0.000164
H	-4.557666	0.813465	0.000083
H	-4.668832	-1.681826	-0.000095
H	-2.535545	-2.990226	-0.000191
H	-0.374512	5.254983	0.889557
H	-0.374872	5.254877	-0.889107
H	1.158710	5.191412	-0.000091

12

12a

WB97(PCM=DMSO)

E(SCF)=-634.27096 **a.u.**
E(SCF+ZPVE)= -634.01789 **a.u.**

```
30
12a WB97 DMSO
N     -1.059575   -0.280985    0.069280
C     -1.224478    0.596666   -1.085930
C     -2.314810    1.582514   -0.934629
C     -0.036478    0.088411    1.061694
C      1.349706    0.036968    0.421462
C     -0.385138    1.411383    1.741925
C      1.750404   -1.150063   -0.210345
C      3.008013   -1.259255   -0.797273
C      3.892772   -0.176044   -0.762896
C      3.505616    1.006756   -0.137778
C      2.240486    1.112459    0.451812
C     -1.866248   -1.361268    0.281858
O     -1.736592   -2.088400    1.275490
C     -2.924247   -1.654264   -0.761128
C     -3.215483    2.380444   -0.825541
H     -1.395933    0.005025   -1.991393
H     -0.276688    1.125159   -1.243008
H     -0.082460   -0.705840    1.813723
H     -0.373030    2.255891    1.041383
H     -1.387764    1.344461    2.177862
H      0.326074    1.625371    2.547382
H      1.062337   -1.996190   -0.240685
H      3.301800   -2.189684   -1.281115
H      4.877067   -0.257848   -1.221407
H      4.186058    1.856505   -0.106158
H      1.959676    2.046416    0.935748
H     -3.541025   -0.774503   -0.974094
H     -2.458049   -1.979283   -1.698773
H     -3.556271   -2.460013   -0.384341
H     -4.012713    3.090381   -0.723392
```

12b

WB97(PCM=DMSO)

E(SCF)= -634.27636 **a.u.**
E(SCF+ZPVE)= -634.02420 a.u.

```
30
12b WB97 DMSO
N     -1.457049    0.136969   -0.026745
C     -1.291430    1.338560   -0.760495
C     -0.270190    2.161730   -0.685167
C      0.711303    3.028288   -0.653286
C     -0.477289   -0.224402    1.013029
C      0.913056   -0.445557    0.412277
C     -0.533070    0.755920    2.188542
C      1.038851   -1.053128   -0.843414
C      2.294476   -1.319137   -1.387527
C      3.451217   -0.984074   -0.679680
C      3.336181   -0.383299    0.573344
C      2.075964   -0.116843    1.115033
C     -2.584961   -0.608124   -0.295143
O     -3.397436   -0.249412   -1.152631
C     -2.809951   -1.887078    0.483849
H     -2.112691    1.547247   -1.440423
H      0.700796    3.869656    0.039749
H      1.575814    2.924440   -1.308883
```

H	-0.793526	-1.196559	1.399761
H	-0.116923	1.735592	1.932059
H	0.022643	0.355788	3.042910
H	-1.575101	0.887450	2.499870
H	0.143219	-1.308812	-1.410039
H	2.369559	-1.787501	-2.367933
H	4.433231	-1.188731	-1.103259
H	4.229856	-0.115298	1.135237
H	2.013096	0.361147	2.090750
H	-3.743078	-2.325242	0.126117
H	-2.895724	-1.695090	1.558840
H	-1.997284	-2.604700	0.325340

12c

WB97(PCM=DMSO)

E(SCF)= -634.27636 **a.u.**
E(SCF+ZPVE)= -634.02420 **a.u.**

30
12c WB97 DMSO

N	-1.243140	-0.343365	0.186013
C	-1.293388	1.007888	-0.008355
C	-0.132966	-0.879455	1.008927
C	1.199421	-0.534357	0.353433
C	-0.290683	-0.426685	2.456551
C	2.174594	0.240091	0.984594
C	3.383528	0.524203	0.339771
C	3.627437	0.036630	-0.942266
C	2.655908	-0.740328	-1.581848
C	1.454691	-1.021647	-0.936463
C	-2.213435	-1.183650	-0.332321
O	-2.165617	-2.397606	-0.142128
C	-3.310565	-0.527900	-1.133172
C	-1.297262	2.207872	-0.155948
C	-1.323314	3.664196	-0.347335
H	-0.264437	-1.965261	0.960959
H	-0.247555	0.665896	2.543763
H	0.498833	-0.857819	3.081660
H	-1.258250	-0.765620	2.841799
H	2.003400	0.631039	1.986163
H	4.133566	1.130544	0.845605
H	4.568620	0.257808	-1.443307
H	2.837984	-1.128624	-2.582879
H	0.698704	-1.628005	-1.438150
H	-3.860180	0.190114	-0.515097
H	-3.988849	-1.305151	-1.488842
H	-2.892000	0.019401	-1.984513
H	-0.406668	4.011770	-0.834603
H	-1.428157	4.184808	0.610029
H	-2.171896	3.942350	-0.981058

13

13a

WB97(PCM=DMSO)

E(SCF)= -709.48889 **a.u.**
E(SCF+ZPVE)= -709.23056 **a.u.**

```
31
13a WB97 DMSO
N     -0.798442    0.026274    0.096380
C     -0.851949    0.864406   -1.096470
C      0.280713    0.252657    1.069784
C      1.632834   -0.036404    0.418505
C      0.157650    1.632537    1.714530
C      1.833776   -1.290954   -0.176540
C      3.049690   -1.613837   -0.772456
C      4.092591   -0.681364   -0.784347
C      3.903891    0.566940   -0.196350
C      2.679960    0.887553    0.402396
C     -1.787417   -0.864512    0.381310
O     -1.826158   -1.581667    1.378377
O     -2.736285   -0.882934   -0.576027
C     -3.806875   -1.819240   -0.366608
C     -1.763686    2.020396   -0.970835
C     -2.509809    2.966757   -0.882540
H     -1.152580    0.260693   -1.958152
H      0.165455    1.222782   -1.293666
H      0.120410   -0.503361    1.845905
H      0.291468    2.442714    0.986747
H     -0.835793    1.737968    2.163683
H      0.907067    1.754255    2.504340
H      1.022085   -2.019855   -0.171665
H      3.187419   -2.593794   -1.227215
H      5.044884   -0.930331   -1.250073
H      4.708475    1.300902   -0.201395
H      2.555191    1.869197    0.856222
H     -4.471423   -1.691751   -1.222360
H     -4.332882   -1.591530    0.564811
H     -3.417274   -2.840712   -0.332797
H     -3.172066    3.806096   -0.800117
```

13b
WB97(PCM=DMSO)

E(SCF)= -709.49158 **a.u.**
E(SCF+ZPVE)=-709.23403 a.u.

```
31
13b WB97 DMSO
N     -1.297435    0.219708   -0.013196
C     -2.461767   -0.396816   -0.530572
C     -2.835814   -1.636066   -0.302131
C     -3.255872   -2.861388   -0.102199
C     -0.412623   -0.533876    0.898323
C      0.996354   -0.668580    0.326067
C     -0.504915   -0.017404    2.333354
C      1.147968   -1.047658   -1.014678
C      2.414128   -1.226698   -1.567808
C      3.554874   -1.034497   -0.782886
C      3.412803   -0.663917    0.553266
C      2.140457   -0.482721    1.104642
C     -1.078523    1.526908   -0.370668
O     -1.799225    2.169195   -1.127598
O      0.023882    2.028107    0.205107
C      0.336656    3.389888   -0.136622
H     -3.059094    0.253998   -1.164021
H     -2.953702   -3.677010   -0.759097
```

```
H     -3.917502   -3.103814    0.729381
H     -0.836156   -1.545779    0.896415
H     -0.053125    0.973980    2.438274
H     -0.003323   -0.709766    3.018484
H     -1.558851    0.041637    2.626879
H      0.261326   -1.198917   -1.632366
H      2.512004   -1.519133   -2.612395
H      4.545972   -1.173621   -1.211995
H      4.294426   -0.510222    1.174119
H      2.052397   -0.185888    2.148253
H      1.251708    3.619108    0.411195
H      0.500728    3.481680   -1.213951
H     -0.474373    4.054328    0.174604
```

13c

WB97(PCM=DMSO)
E(SCF)=-709.49157 a.u.
E(SCF+ZPVE)=-709.23403

```
31
13c WB97-DMSO
N     -0.966618    0.006758    0.525369
C     -1.124448    1.267170    0.024898
C      0.227934   -0.286295    1.359437
C      1.474339   -0.335179    0.486078
C      0.305238    0.686359    2.531534
C      2.219240    0.812167    0.189809
C      3.335440    0.736025   -0.645729
C      3.721682   -0.488896   -1.192597
C      2.986088   -1.639038   -0.900240
C      1.870020   -1.559508   -0.066340
C     -1.791935   -1.030002    0.138348
O     -1.637090   -2.191068    0.489770
O     -2.783048   -0.609134   -0.655880
C     -3.696473   -1.632074   -1.095212
C     -1.208345    2.402454   -0.381002
C     -1.326769    3.776660   -0.887054
H      0.046803   -1.292741    1.746947
H      0.393526    1.726244    2.198042
H      1.177416    0.447001    3.149147
H     -0.595992    0.600012    3.148081
H      1.932369    1.775132    0.610657
H      3.906126    1.636716   -0.867463
H      4.594539   -0.547375   -1.841119
H      3.283030   -2.599636   -1.318706
H      1.294558   -2.457833    0.160414
H     -4.421440   -1.116009   -1.725685
H     -3.161372   -2.394372   -1.668176
H     -4.191923   -2.090119   -0.234674
H     -1.614486    4.469390   -0.089477
H     -0.378481    4.115871   -1.316711
H     -2.089315    3.822976   -1.671557
```

14a

WB97(PCM=DMSO)
E(SCF)= -728.92222 a.u.
E(SCF+ZPVE)= -728.62265 a.u.

```
35
14a WB97 DMSO
N      0.639916   -0.599686   -0.295170
C      0.550735    0.341617   -1.351471
C      1.033112    1.564546   -1.361060
C      1.565106    2.759188   -1.439040
C     -0.567663   -1.419965   -0.059525
C     -1.805540   -0.544266    0.133600
C     -0.696635   -2.485952   -1.147163
C     -2.932674   -0.635202   -0.686720
C     -4.041705    0.190749   -0.470174
C     -4.034879    1.116900    0.570308
C     -2.910663    1.215170    1.397011
C     -1.808645    0.392084    1.179074
C      1.561399   -0.468243    0.750265
O      1.236359   -0.685185    1.928059
N      2.828273   -0.106576    0.408830
C      3.433835   -0.356310   -0.891981
C      3.755161    0.241571    1.474995
H     -0.003233   -0.010726   -2.221578
H      1.018381    3.643662   -1.113829
H      2.573900    2.905421   -1.827221
H     -0.378623   -1.926407    0.892309
H     -0.856172   -2.056032   -2.143759
H     -1.538449   -3.153433   -0.931077
H      0.219159   -3.085387   -1.176550
H     -2.961653   -1.351965   -1.505914
H     -4.911143    0.105468   -1.120730
H     -4.898029    1.759128    0.739233
H     -2.895962    1.934906    2.214575
H     -0.930026    0.464578    1.821171
H      2.780359   -0.979407   -1.505887
H      4.380063   -0.890145   -0.739750
H      3.639298    0.581047   -1.425140
H      3.210422    0.686330    2.309962
H      4.300256   -0.641414    1.837765
H      4.477592    0.968375    1.087622
```

14b

WB97(PCM=DMSO)
E(SCF)= -728.92853 a.u.
E(SCF+ZPVE)= -728.62993 a.u.

```
35
14b WB97 DMSO
N     -0.868597   -0.430396   -0.028123
C     -0.770975   -0.635631    1.368320
C      0.332865   -0.828297    2.057174
C      1.383928   -1.069296    2.801118
C      0.214874   -0.885057   -0.914739
C      1.485026   -0.066365   -0.680795
C      0.397139   -2.402206   -0.847004
C      2.756516   -0.646125   -0.708769
C      3.901777    0.134490   -0.528267
C      3.791750    1.508568   -0.318641
C      2.525367    2.098959   -0.297932
C      1.386332    1.316905   -0.480959
C     -2.114375   -0.182006   -0.603652
O     -2.395672   -0.603442   -1.733319
N     -3.011638    0.523067    0.156686
C     -4.378687    0.599181   -0.339559
C     -2.623239    1.640240    1.016025
H     -1.725088   -0.659137    1.892202
H      1.755618   -2.084603    2.942873
H      1.930673   -0.261403    3.287437
```

H	-0.141862	-0.645635	-1.922398
H	0.811992	-2.725331	0.114799
H	1.064273	-2.746140	-1.644771
H	-0.575881	-2.884172	-0.990386
H	2.868437	-1.718518	-0.857567
H	4.882937	-0.338201	-0.547588
H	4.683856	2.115946	-0.173423
H	2.424782	3.171735	-0.138292
H	0.401682	1.784576	-0.458898
H	-4.487046	1.378809	-1.107528
H	-5.038562	0.836903	0.501493
H	-4.674623	-0.360760	-0.767467
H	-1.537166	1.715258	1.096502
H	-3.001099	2.576753	0.584127
H	-3.045694	1.522612	2.021569

14c

WB97(PCM=DMSO)
E(SCF)= -728.93041 a.u.
E(SCF+ZPVE)= -728.63186 a.u.

35
WB97 DMSO

N	-0.715917	-0.250159	0.312816
C	-0.601238	1.103620	0.436805
C	-0.459543	2.298730	0.560145
C	-0.305839	3.754516	0.692252
C	0.385177	-1.070098	0.870675
C	1.683216	-0.703934	0.157579
C	0.424877	-0.970216	2.392309
C	1.731619	-0.798079	-1.241065
C	2.896181	-0.490029	-1.939003
C	4.039783	-0.080032	-1.245368
C	4.001997	0.017025	0.143607
C	2.828839	-0.292630	0.841289
C	-1.975410	-0.855168	0.149028
O	-2.213354	-1.951772	0.667528
N	-2.888607	-0.170073	-0.590470
C	-2.543164	0.748764	-1.671857
C	-4.255149	-0.673432	-0.608780
H	0.729885	4.048748	0.492925
H	-0.946189	4.271210	-0.030733
H	-0.574456	4.097529	1.696835
H	0.127559	-2.099510	0.598529
H	0.629464	0.056874	2.719318
H	1.197350	-1.628221	2.805718
H	-0.544164	-1.280091	2.796755
H	0.839961	-1.113097	-1.785002
H	2.914946	-0.570311	-3.024985
H	4.952551	0.161525	-1.787881
H	4.885993	0.336978	0.693469
H	2.820743	-0.207870	1.926552
H	-2.813534	1.782157	-1.423344
H	-3.089866	0.443260	-2.572250
H	-1.474047	0.705306	-1.891083
H	-4.500268	-1.116865	0.358214
H	-4.932134	0.165086	-0.802465
H	-4.393355	-1.431128	-1.392917
